# Genome wide transcriptomic analysis of the soil ammonia oxidizing archaeon *Nitrososphaera viennensis* upon exposure to copper limitation

**DOI:** 10.1038/s41396-020-0715-2

**Published:** 2020-07-14

**Authors:** Carolina Reyes, Logan H. Hodgskiss, Melina Kerou, Thomas Pribasnig, Sophie S. Abby, Barbara Bayer, Stephan M. Kraemer, Christa Schleper

**Affiliations:** 1 Department of Environmental Geosciences, Centre for Microbiology and Environmental Systems Science, University of Vienna, Althanstrasse 14, UZA2, 1090 Vienna, Austria; 2 Department of Functional and Evolutionary Ecology, Archaea Biology and Ecogenomics Unit, University of Vienna, Althanstrasse 14, UZA1, 1090 Vienna, Austria; 3 Environmental Science Research Network (ESRN), Faculty for Geosciences, Geography and Astronomy, University of Vienna, Althanstrasse 14, UZA2, 1090 Vienna, Austria; 4 University Grenoble Alpes, CNRS, Grenoble INP, TIMC-IMAG, 38000 Grenoble, France; 5 Department of Limnology and Oceanography, Division of Bio-oceanography, University of Vienna, Althanstrasse 14, UZA1, 1090 Vienna, Austria; 6 Present Address: Department of Ecology, Evolution and Marine Biology, University of California Santa Barbara, Santa Barbara, CA 93106-9620 USA

**Keywords:** Archaeal genomics, Archaeal physiology

## Abstract

Ammonia-oxidizing archaea (AOA) are widespread in nature and are involved in nitrification, an essential process in the global nitrogen cycle. The enzymes for ammonia oxidation and electron transport rely heavily on copper (Cu), which can be limited in nature. In this study the model soil archaeon *Nitrososphaera viennensis* was investigated via transcriptomic analysis to gain insight regarding possible Cu uptake mechanisms and compensation strategies when Cu becomes limiting. Upon Cu limitation, *N. viennensis* exhibited impaired nitrite production and thus growth, which was paralleled by downregulation of ammonia oxidation, electron transport, carbon fixation, nucleotide, and lipid biosynthesis pathway genes. Under Cu-limitation, 1547 out of 3180 detected genes were differentially expressed, with 784 genes upregulated and 763 downregulated. The most highly upregulated genes encoded proteins with a possible role in Cu binding and uptake, such as the Cu chelator and transporter CopC/D, disulfide bond oxidoreductase D (*dsbD*), and multicopper oxidases. While this response differs from the marine strain *Nitrosopumilus maritimus*, conserved sequence motifs in some of the Cu-responsive genes suggest conserved transcriptional regulation in terrestrial AOA. This study provides possible gene regulation and energy conservation mechanisms linked to Cu bioavailability and presents the first model for Cu uptake by a soil AOA.

## Introduction

Ammonia-oxidizing archaea (AOA) perform the first and rate-limiting step of nitrification along with ammonia-oxidizing bacteria (AOB) and comammox [1–3]. AOA are ubiquitous and abundant in most aerobic environments [4, 5]. Cultivated AOA strains belonging to the class *Nitrososphaeria* of the phylum Thaumarchaeota [6] represent some but not all of the currently recognized lineages of AOA based on environmental gene sequences [7].

A common feature of AOA is the central role of copper (Cu) in their metabolism. Copper is used as a cofactor to many key proteins in AOA including the ammonia monooxygenase (AMO), which performs the energy-gaining step of ammonia oxidation, plastocyanins, which are encoded in high numbers by AOA and used presumably as electron carriers, and multicopper oxidases, whose role although conserved in AOA remains unclear [8, 9]. While the AMO of AOB also uses Cu as a cofactor, mostly heme-containing proteins such as hydroxylamine oxidoreductase (HAO) and cytochrome C_M_552 (CycB) make up the bacterial ammonia oxidation electron transport chain [10]. AOA, in contrast, lack homologs for both HAO and CycB. The enzyme replacing HAO in AOA has yet to be determined, while Cu containing plastocyanins are hypothesized to act as electron carriers in place of CycB [8]. Therefore, AOA should depend more highly on Cu containing enzymes than their bacterial counterparts, which would necessitate elaborate regulatory mechanisms to respond to rapidly changing local concentrations of this metal. Indeed, the marine AOA *Nitrosopumilus maritimus* SCM1, with Cu adsorbed on its surface, exhibited exothermic enthalpies in contrast to neutral or endothermic enthalpies for AOB, suggesting a higher surface selectivity of AOA for Cu [11].

The abundance of Cu in the Earth’s crust is significantly lower than that of other biologically important metals [12]. In terrestrial environments, its bioavailability is influenced primarily by organic matter content and pH [13]. Recently, Gwak et al. [14] showed that mixtures of organic compounds like yeast extract depleted Cu in culture experiments with AOA and AOB. However, only AOA growth was inhibited by reduced Cu bioavailability. These results support the idea that Cu is a more important trace element for AOA than AOB.

While the mechanisms by which different AOA clades take up Cu from their environment are not well understood, genomic analysis of the transporter complement of six different AOA, including the soil archaeon *Nitrososphaera viennensis* (EN76) [15, 16], showed that orthologs of multicopper oxidases and ZIP family permeases are conserved among AOA and could have a role in making Cu more bio-available and in Cu uptake respectively [9]. In addition, all known AOA harbor proteins belonging to the copper resistance CopC and CopD families, typically involved in Cu resistance mechanisms [17–20]. In general, Cop proteins are suggested to import Cu into cells [21, 22], to export Cu out of cells [23], and to bind Cu in the periplasm [24].

The effects of Cu-limitation on cell physiology, metabolism, and uptake have been studied on pure cultures of *N. maritimus* SCM1 [25, 26] and *N. viennensis* (EN76) [13]. These studies support the idea that Cu is essential for AOA and show that as Cu concentration becomes limiting, ammonia oxidation activity and cell growth decrease. While Qin et al. [26] provides a first outline to the Cu-limitation transcriptomic response in marine AOA, the corresponding mechanisms in terrestrial lineages remain unexplored. Terrestrial AOA generally have larger genomes that have evolved under different selection pressures and exhibit different capabilities than the streamlined marine AOA [9].

The aim of this study was to identify genes potentially involved in Cu-uptake and to identify physiological and regulatory responses in *N. viennensis* under Cu-limiting conditions. As such, a genome-wide transcriptomic analysis of *N. viennensis* in response to Cu-limiting conditions was performed and is presented along with supporting proteomic and quantitative PCR results. While the reaction of *N. viennensis* markedly differs from that of *N. maritimus* with respect to possible Cu-sequestration, conserved regulatory motifs in downregulated genes show conservation among related soil strains.

## Methods

Detailed cultivation procedures, measurement of ammonia oxidation, RNA extraction, protein extraction, quantitative reverse transcription PCR (RT-qPCR), sequencing methods, sequencing analysis, proteomic methods and analysis, motif analysis, and phylogenetic analysis are described in the Supplementary material.

### Cu-limiting conditions for differential gene expression

The Cu chelator TETA (1,4,8,11-tetraazacyclotetradecane N, Nʹ, N″, N‴-tetraacetic acid hydrochloride hydrate) with selective affinity for Cu^2+^ was used to induce Cu limitation as previously described [13]. Five replicate 500 mL cultures were each inoculated either with a Cu-limited (Cu12) or a standard *N. viennensis* culture to establish Cu-limited and Cu-replete cultures respectively. It was estimated using the PhreeqC program [27] that Cu-limited cultures Cu10 and Cu12 contained 6 × 10^−15^ mol/L and 8 × 10^−16^ mol/L of bioavailable Cu^2+^, respectively [13].

### RNA and proteome analysis

Five replicate cultures of each growth condition of *N. viennensis* were harvested by filtration followed by extraction of RNA from the filters. Extracted RNA (cDNA) of each sample was sequenced by the Vienna Biocenter Facility [VCBF], Vienna, Austria, using an Illumina HiSeq2500 instrument. Raw read files were processed and then analyzed for differential expression using DESeq2 [28]. The Benjamini Hochberg DESeq2 default step was used to control for false discovery rate (FDR). Genes with an adjusted *p* value (*p-*adj) < 0.01 were considered differentially expressed.

**Fig. 1:**
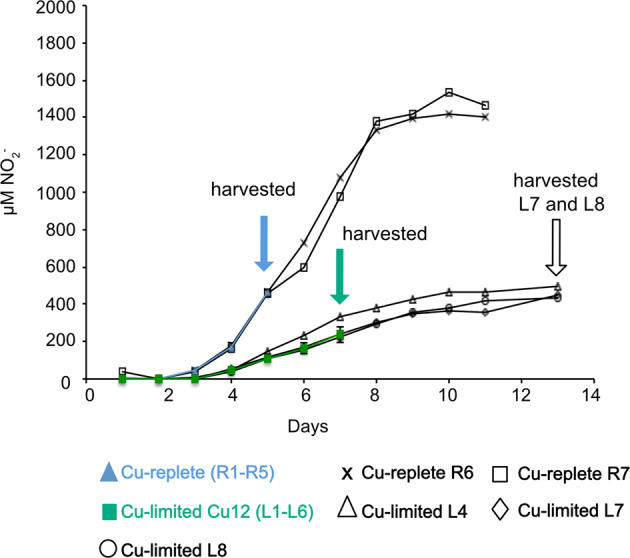
Nitrite concentrations in batch cultures of *N. viennensis* grown in 500 mL in 1 L polystyrene bottles under Cu-replete and Cu-limited (Cu12) conditions. Cu replete cultures, labeled (R1–R5) refer to the five biological replicates R1, R2, R3, R4, and R5 that were harvested on day 5. Cu limited cultures, labeled (L1–L3, L5-L6) refer to the five biological replicates as L1, L2, L3, L5, and L6 that were harvested on day 7. Solid arrows indicate when cultures were harvested for RNA extraction. Open arrow indicates when cultures were harvested for protein extraction. The data and error bars for Cu-replete and Cu-limited cultures represent the average and standard deviation of biological replicates.

Proteomic and RT-qPCR analyses were performed on two additional Cu-limited cultures (L7 and L8) that were harvested on Day 13 (Fig. 1). While the proteomes from two Cu-limited cultures verified the presence of multicopper oxidases, further critical analysis of the proteomic data was not carried out due to the lack of more biological replicates and the lack of proteome data from the control cultures.

All raw sequence runs from the RNA-Seq analysis were deposited in the DNA Databank of Japan (DDBJ) under the accession number DRA008627. The mass spectrometry proteomics data have been deposited to the ProteomeXchange Consortium via the PRIDE [29] partner repository with the dataset identifier PXD018461 and 10.6019/PXD018461.

## Results and discussion

### Growth response under Cu-limiting conditions

Initial growth tests with *N. viennensis* were performed in the presence and absence of TETA in order to determine if this Cu chelator affects growth of the organism. TETA alone did not have a toxic effect on cell growth, as cultures supplemented with TETA and excess Cu produced nitrite concentrations comparable to the Cu-replete cultures (Fig. S1). When TETA was applied to Cu-limited cultures, a decreased ability to oxidize ammonia compared to Cu-replete cultures was observed, while re-introduction of Cu to Cu-limited (Cu10) cultures restored their ability to produce nitrite comparable to the Cu-replete culture (Fig. S1) [13]. These results demonstrate that TETA can be used to chelate Cu and induce Cu-limitation in *N. viennensis* and that Cu-limited cells retain the ability to oxidize ammonia following Cu addition. Metabolic recovery following Cu starvation has also been observed in studies with *N. maritimus* [25], where cells that were starved of Cu for up to 6 days recovered within 24 h of Cu addition.

For the transcriptome analyses, under Cu-limiting versus Cu-replete conditions, five replicates each were grown in parallel. At the time cultures were harvested for RNA extraction, Cu-replete cultures had produced 436–493 µM nitrite and Cu-limited (Cu12) cultures had produced 202–334 µM nitrite (Fig. 1).

### RNA sequencing analysis

**Fig. 2:**
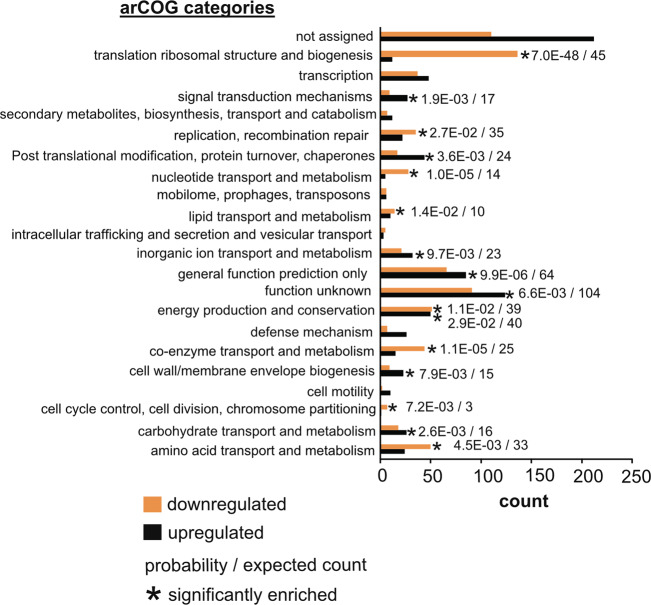
Bar charts showing up and downregulated genes assigned to functional groups in Cu-limited samples. Genes were assigned to functional categories based on their archaeal clusters of orthologous group (arCOG) categories. A *p* value < 0.05 cutoff was used to determine significance (see Supplemental Methods).

RNA sequencing results are reported in Table S1. To analyse variation in gene expression between Cu-replete and Cu-limited conditions and between replicates of each condition a PCA analysis was performed. PCA results showed that the five replicates of each condition grouped together (Fig. S2) thus demonstrating high reproducibility. One principle component (PC1) could explain ~51 % of the variance between the Cu-limited and Cu-replete cultures. Comparison of protein-coding genes between Cu-limited and Cu-replete samples resulted in 1,547 differentially expressed genes out of 3,180 genes identified from reads mapped to the genome (Dataset S1) (Benjamini Hochberg adjusted *p* value cut-off of 0.01). After careful evaluation of the data a *p* value cut-off of 0.01 was chosen instead of the more commonly used 0.05 to define deferentially expressed genes, which allowed for a better focus of the results of the DESEq2 analysis. Because of the low adjusted *p* values and therefore high statistical reproducibility, genes meeting this criteria were considered even if they showed small changes (in terms of the log_2_ fold change) between the two conditions. This is in line with previous archaeal studies that have identified and/or observed genes of physiological significance with a focus on a *p* value cut-off irrespective of the low log_2_ fold changes [30, 31]. Of the differentially expressed genes, 784 were upregulated (24.7 % of detected genes) and 763 were downregulated (24.0% of detected genes) in Cu-limited cultures. Overall, these results showed that 50% of the differentially expressed genes were upregulated in response to Cu-limitation compared to 30% of genes upregulated in Cu-limited *N. maritimus* cultures [26]. Next, to gain insight into the general biological function of differentially expressed genes in Cu-limited samples, a hypergeometric test was used to determine which arCOG categories were significantly enriched (*p* value < 0.05) in up and downregulated groups. The hypergeometric test revealed enriched arCOG functional categories within the upregulated group including: inorganic ion transport and metabolism; signal transduction mechanisms; and posttranslational modification, turnover, and chaperones (Fig. 2). In the downregulated group, enriched categories included: nucleotide transport and metabolism; replication, recombination and repair; and translation/ribosomal structure and biogenesis (Fig. 2). An enrichment of the inorganic ion transport and metabolism category within the upregulated group suggests that the Cu-limited cultures directly responded to Cu limitation on a transcriptional level. Furthermore, the enrichment of translation, replication, and nucleotide metabolism genes within the downregulated group denote a global downregulation of genes associated with growth. These results and those of the growth experiments, indicate that the cells were unable to effectively replicate and oxidize ammonia to nitrite, a proxy for growth [15], due to insufficient levels of Cu [26].

**Fig. 3:**
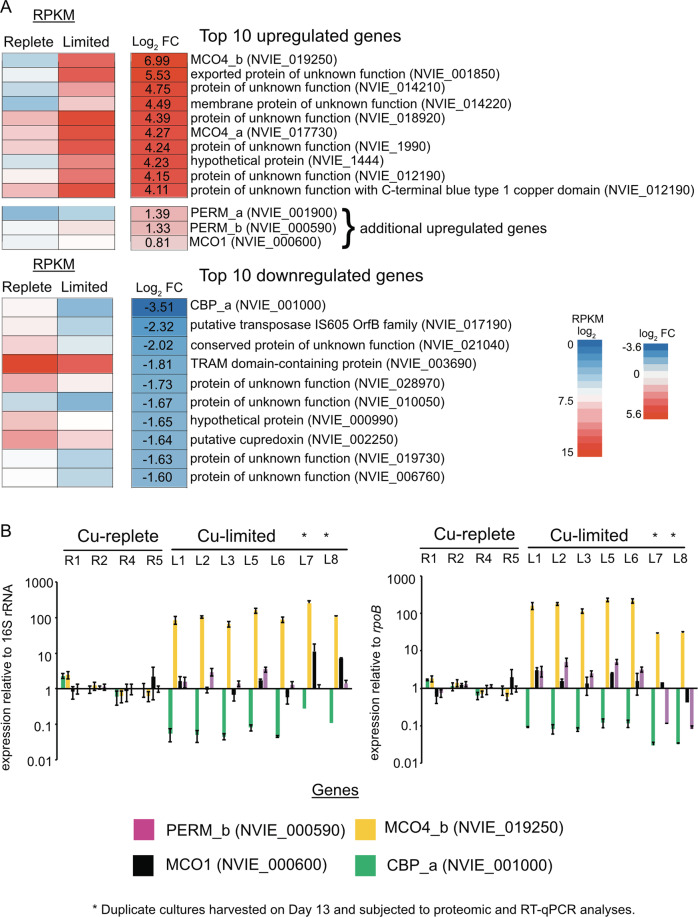
Top upregulated and downregulated genes. **a** Top 10 up and downregulated genes and additional upregulated genes. A *p* value < 0.01 cutoff was used to determine significance. **b** qPCR results of select up and downregulated genes relative to the 16 S rRNA and *rpoB* gene in biological replicates of Cu limited and Cu replete cultures. A logarithmic scale was used for the y-axis. Samples L7* and L8* are Cu-limited cultures collected on Day 13 (see Fig. 1) and subjected to proteomic and RT-qPCR analysis.

The top upregulated and downregulated genes are shown in Fig. 3a. In Cu-limited cultures, the multicopper oxidase gene MCO4_b (NVIE_019250), was the most upregulated gene (log_2_ fold change (FC) of 6.99) and CBP_a (NVIE_001000), a gene encoding a putative surface-associated Ca^2+^ binding protein, was the most downregulated (log_2_ FC −3.51).

### Genes encoding MCOs and associated proteins

Cu-limitation induced the expression of several proteins possibly involved in Cu uptake and transport. Previously, the two domain MCOs (2d MCOs) [32, 33] of *N. viennensis* were phylogenetically compared to other archaeal and bacterial 2d MCOs and were divided into four separate lineages (MCO1-4) [9]. MCOs belonging to lineage 1 (MCO1) were only found in thaumarchaeal genomes, whereas MCOs belonging to lineage 4 (MCO4) were only present in the order Nitrososphaerales and the genera *Nitrosopumilus* and *Nitrosarchaeum* [9]. MCOs of the type encoded by MCO1 and MCO4 genes contain three types of Cu centers (T1–T3), while MCO4 has an extra blue copper domain [9].

In this study, two highly upregulated MCO4 genes, MCO4_a (NVIE_017730; log_2_ FC 4.27) and MCO4_b (NVI_019250; log_2_ FC 6.99), and one moderately upregulated MCO1 (NVIE_000600; log_2_ FC of 0.81) were identified. In addition, a putative zinc/iron permease gene adjacent to MCO1, PERM_b (NVIE_000590; log_2_ FC 1.33), and another zinc/iron permease, PERM_a (NVIE_001900; log_2_ FC 1.39), were also differentially expressed. Both MCO4s and the MCO1 were also detected in the proteome of the two tested Cu-limited cultures (Dataset S1). The two zinc/iron permeases were absent from the proteome, likely attributed to the protein extraction method that was not specifically adapted for membrane proteins. In RT-qPCR, general trends of selected up and downregulated genes were also confirmed across all copper limited samples used for transcriptomics or proteomics: MCO4_b (NVIE_019250) was highly expressed, and PERM_b (NVIE_000590) and MCO1 (NVIE_000600) were moderately expressed, whereas CBP_a (NVIE_001000) was under-expressed relative to the housekeeping genes 16 S rRNA and *rpoB* in Cu-limited cultures (Fig. 3b; see Table S2 for primers).

Comparison of MCO1 and MCO4 with other 2d MCOs from bacteria (Fig. S3) suggests that the T1 Cu site in these MCOs are coordinated by two histidines, a cysteine and either a methionine (in MCO4_a; NVIE_017730) or leucine (in MCO1; NVIE_000600 and MCO4_b; NVIE_019250). In bacteria, the T2/T3 Cu sites of MCOs can reduce oxygen to water and oxidize organic (e.g., siderophores, phenolic compounds) or inorganic (e.g., Cu^+^, Fe^2+^) electron acceptors [17, 32] via the T1 Cu site [34]. MCOs in archaea could use a similar mechanism to oxidize Cu^+^ to Cu^2+^, for example to make Cu available to cells as discussed below.

Interestingly, genes for proteins requiring Cu (like MCOs) were upregulated while the organism was under Cu limitation. One possible explanation for this observation is that Cu limitation does not affect transcription of these MCO genes directly, but rather their stability and proper function following translation. This is true of MCOs with ferroxidase activity in Cu-deficient mammals [35]. Similarly, in expression studies of MCOs in the yeast *Candida albicans*, some MCO transcripts were upregulated under Cu limitation while others were unaffected [36]. This implies the cell recognizes that the proteins are defective and increases transcription to make new proteins while attempting to remedy the situation. In this scenario, improperly folded proteins rather than direct Cu-limitation would be inducing a transcriptional response.

Another possibility is that MCOs make Cu more bio-available to cells. In *Saccharomyces cerevisiae*, the MCO Fet3p oxidizes iron which is then transported into the cell via a permease [37]. Perhaps a similar strategy is employed for Cu transport into AOA cells using MCOs and permeases, whose genes are directly linked in the genome (i.e., NVIE_000600 and NVIE_000590; NVIE_026950 and NVIE_026940). In addition, *Escherichia coli* has an MCO, CueO, that has been shown to oxidize Cu^+^ to Cu^2+^ [38]. MCOs in AOA could potentially oxidize Cu^+^ to Cu^2+^ which in turn could be more readily transported by the ZIP permeases. However, the hypothesis that these MCOs and permeases are involved in Cu transport has to be experimentally validated.

### Genes encoding Cu-containing Cop proteins and their possible regulation

A putative Cu-storage protein, Csps (NVIE_028200), was identified and upregulated in *N. viennensis* under Cu limitation. This Csps contains 18 cysteine residues (15.3% of the amino acid sequence) and is similar (28.44% identity; BLASTP 2.10.0+ analysis [39, 40]) to a Cu-storage protein found in *Methylosinus trichosporium* OB3b, MtCsp1. A study of *M. trichosporium* OB3b showed that under Cu limitation MtCsp1 stores Cu^+^ for use by its particulate methane monooxygenase, allowing cells to grow on methane when Cu is limiting [41]. Perhaps a similar strategy is used by *N. viennensis* to gather enough Cu for its AMO complex.

**Fig. 4:**
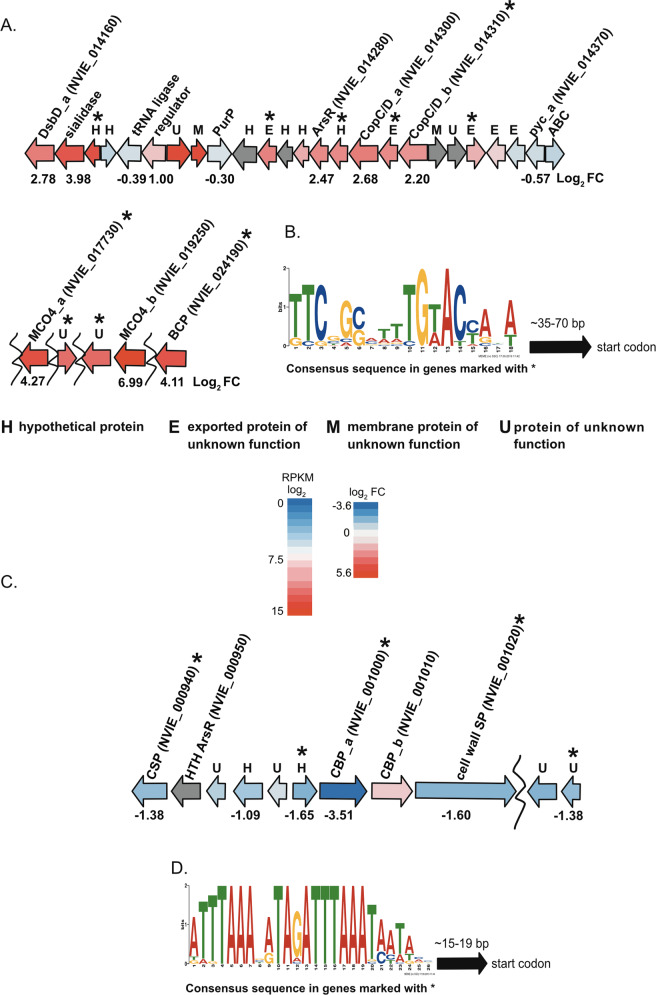
Upregulated and downregulated gene clusters. **a** Upregulated gene clusters along with their gene expression information. Curved line indicates genes are in another gene cluster relative to the other genes. Gene arrow colors represent log_2_ fold change of genes in copper limited cultures. Gray arrows are not differentially expressed. **b** A motif consensus sequence found in certain upregulated genes marked with an asterix (*) in **a**. DsbD, disulfide bond oxidoreductase D family protein; sialidase, putative sialidase neuraminidase family protein; tRNA ligase, putative threonyl/alanyl tRNA ligase; regulator, putative transcriptional regulator; PurP, 4-carboxamide-1-(beta)-D-ribofuranosyl 5ʹmonophosphate ligase; ArsR, transcriptional regulator ArsR; pycA, putative plastocyanin Cu containing protein/azurin family; ABC, putative ABC transporter; MCO, multicopper oxidase; BCP, protein of unknown function with C-terminal blue type(1) copper domain. Locus tag information for all genes in this figure can be found in Dataset S1. **c** Gene clusters of top downregulated genes that were found to harbor a motif sequence along with their gene expression information. Curved line indicates genes are in another gene cluster relative to the other genes. Gene arrow colors represent log_2_ fold change of genes in copper limited cultures. Gray arrows are not differentially expressed. **d** A motif consensus sequence found in certain upregulated genes marked with an asterix in **c**. PUA, PUA domain containing protein; Lrp, transcriptional regulator Lrp family; psmB1, psmB1 proteosome subunit beta; CSP, cell surface protein w/DUF 11 domain; HTH-ArsR, hypothetical protein w/HTH ArsR; CBP, cell surface-associated Ca^2+^ binding domain protein aemolysin type; cell wall SP, putative cell wall surface anchor family protein. Gene locus information for all genes in figure can be found in Dataset S1.

Cop proteins also seem to play a role in Cu limitation, as several of their genes were upregulated including: two *copC/D* genes, *copC/D_a* (NVIE_014300) and *copC/D_b* (NVIE_014310). Both proteins are fusions of CopC and CopD domains, and their genes together with neighboring genes form part of a larger cluster comprising 25 genes of which 15 were upregulated (Fig. 4a). In bacteria, CopC is a hypothetical chaperone located in the periplasm that brings Cu to CopD, an inner membrane protein, perhaps helping to transport Cu into the cytoplasm [21]. Alignment of the *N. viennensis* CopC/D amino acid sequences to CopC representative sequences from the Lawton et al. 2016 study [21] showed they were most similar to CopC from *M. trichosporium* OB3b (*Mst*-CopC, Fig. S4). *Mst*-CopC has one binding site for Cu^2+^ (Fig. S4). CopCs similar to *Mst*-CopC, with one Cu^2+^ binding site, are the most common type of CopC proteins [21]. In these CopC proteins, a methionine rich region that would bind Cu^+^ is missing [21]. The missing methionine residues have been noted to occur in species (including *N. viennensis*) that are missing *copA* and *copB*, encoding for a multicopper oxidase and outer membrane protein respectively, from the classical *copABCD* operon [42]. Furthermore, Gram-positive bacteria were observed to not only lack the methionine residues, but to also contain a fusion of CopC/D [42]. This fusion was suggested to be relevant for Gram-positive bacteria due to the absence of a periplasm (defined as a space between two membranes) when compared to Gram-negative bacteria [42]. Therefore, the lack of a true periplasm in Thaumarchaeota might also account for the CopC/D fusion protein that is conserved across the phylum (Fig. S5).

CopC and CopD are primarily involved in Cu resistance, but some exceptions are known. In *Pseudomonas fluorescens* SBW25, an organism also lacking CopA and CopB homologs, overexpression of *copCD*, resulted in hypersensitivity to Cu [24] supporting the idea that CopCD is involved in Cu uptake in some microorganisms. In two marine AOA species, the expression of CopC/D was increased as a response to oxidative stress, and was suggested to represent a strategy to reduce oxidative damage through copper accumulation [43].

The expression of CopCD under Cu limitation was also proposed to be regulated by CopRS [24], a two-component regulatory system that is known to control *copABCD* operons [44]. Indeed, an upregulated potential two-component system under Cu limitation was found in *N. viennensis* (NVIE_030040, log_2_ FC 1.77; NVIE_030060, log_2_ FC 1.51) with NVIE_030040 sharing a 27.97% identity to CopR from *P. fluorescens* SBW25 (PFLU_1576 or cusR, NCBI accession number CAY47824.1) [39, 40]. Potentially, these two proteins could act as CopR and CopS respectively. If so, a regulatory system induced by Cu limitation, similar to that proposed in *P. fluorescens* SBW5, is likely.

Alternatively, it was suggested that in the bacterium *Corynebacterium glutamicum* CopC could participate in the assembly of cytochrome *aa*_*3*_ oxidase (complex IV) as an intermediate Cu chaperone before Cu is incorporated into the cytochrome oxidase [45]. This could also be true for *N. viennensis* as the cytochrome oxidase in Thaumarchaeota is classified as type *aa*_*3*_ [46, 47]. Whether or not Cu needs to be transported into the cell before incorporation (as for *cbb*_*3*_ oxidase in the bacterium *Rhodobacter capsulatus* [48, 49]) or whether this takes place in the (pseudo-)periplasm (as in the model for cytochrome *aa*_*3*_ oxidase in *Paracoccus denitrificans* [50]) is unclear.

Genomic organization in *N. viennensis* revealed that the Cop genes are spread out in the genome with CopC/D genes residing in one genomic region, CopA1 (NVIE_008380) in another, and CopA2 (NVIE_012900), CopZ (NVIE_012910), and one of three CopT genes (NVIE_012920) residing in a third genomic region (Fig. S6). In *N. viennensis*, CopA1 and CopA2 are P1B-type (also known as CTPx-type) ATPase proteins (Fig. S7a, b respectively) and not the CopA-MCO discussed above. This discrepancy is a result of redundant naming in the literature. In *N. viennensis*, CopA1 and CopA2 refer exclusively to P1B-type ATPase proteins. A number of studies have shown that P1-type ATPases can function as exporters and importers of ions [20]. CopZ and CopT are located in the same gene cluster as CopA2 (Fig. S6). CopZ could deliver Cu^+^ to CopA1 or CopA2 [51] for export. CopT is similar (38% identity) to a novel archaeal transcriptional regulator (SO2652) that is hypothesized to control the expression of Cop genes in *Sulfolobus solfataricus* P2 [19]. Of these Cop genes in *N. viennensis*, only the CopC/D genes and the CopA2 gene were differentially upregulated under Cu-limitation (Dataset S1). This is unusual for CopA2 as it is assumed to be an exporter, but perhaps it is constitutively expressed to respond to sudden fluctuations in Cu availability. How these Cop genes are transcriptionally regulated remains an open question.

### Shared promoter motifs in differentially upregulated genes

To investigate possible regulatory mechanisms of the upregulated genes, upstream regions from translational start sites were analyzed for shared motifs. Initially, no motifs were identified in the upstream regions of the top 25 upregulated genes. The analysis was repeated focusing on 15 upregulated genes known or suspected to be involved in Cu metabolism. These included the upregulated genes with a log_2_ FC > 1.5 found in close proximity to the two CopC/D genes (NVIE_014300 and NVIE_014310) in the genome, upregulated genes next to the highly upregulated MCO4_b (NVIE_019250), and genes specifically associated with Cu from the top 25 upregulated genes (see Dataset S1 for a full list of genes).

A motif with a consensus sequence of 5ʹ-TTCSGSMTTTGWACYANT-3ʹ was found 12 times starting at position −35 to −70 with respect to the potential translational start codon of the respective gene (Fig. 4b). In particular, it was found twice in front of one of the CopC/D genes, *copC/D_b* (NVIE_014310) (Fig. S8a). For some of the analyzed genes, the motif was found in the upstream region on the opposite strand in reference to the gene orientation (Fig. S8a). This was the case for an upregulated exported protein of unknown function near the CopC/D genes (NVIE_014340), a protein of unknown function (NVIE_019240) that directly follows the highly upregulated MCO4_b (NVIE_019250), a highly upregulated gene of unknown function near the *copC/D* region of the genome (NVIE_1444), and MCO4_a (NVIE_017730). Regarding NVIE_1444, the motif was also repeated. A motif recruiting a transcriptional activator on the opposite strand in reference to the transcribed gene was previously observed upstream of the *malZ* gene in *E. coli*. [52]. Once found, the specific motif sequence was screened against the top 25 upregulated genes. This resulted in the additional discovery of the motif in the second most upregulated gene (NVIE_001850, log_2_ FC 5.53), an exported protein of unknown function. The presence of this motif in various upregulated genes could indicate that it is involved in transcription under Cu limitation.

Most of the upregulated genes with the motif, or in a putative operon with the motif, encode hypothetical or unknown proteins. An ArsR family transcriptional regulator (NVIE_014280), a family known to be involved in metal stress [53], was one of the upregulated genes near the CopC/D cluster and is presumably co-transcribed with NVIE_014290 containing the identified motif (Fig. 4a). Another gene, NVIE_014160 (DsbD_a), a disulfide bond oxidoreductase D (DsbD) family protein, was located in a putative operon starting with NVIE_1444. DsbD family proteins shuttle electrons from the cytoplasm to the periplasm to reduce oxidized disulfide bonds and/or to adjust the redox status of prokaryotic cells. Since further counterparts of the redox system are missing, the upregulation of *dsbD* could be a cellular response to rectify damage to disulfide bonds induced by a lack of Cu rather than a mechanism to adjust the cell’s redox state. Alternatively, DsbD, like its homolog ScsB, could bind Cu^+^ before passing it to a Cu chaperone in the periplasm [54, 55], thus sequestering Cu rather than removing it (as in *S. enterica* [56]).

### Downregulated genes

A common motif was also searched for in the upstream region of the top 25 downregulated genes. A motif with a sequence of 5ʹ-RATTTAAABATAGATTTAAATAATAAS-3ʹ was identified in five of the top 25 downregulated genes (Fig. 4c, d and S8b): NVIE_001000 (the most downregulated gene), NVIE_0009400, NVIE_0009900, NVIE_001020, and NVIE_019740. NVIE_019740 appears to form an operon with NVIE_019730, which is also in the top 25 downregulated genes. Therefore, only the upstream sequence of NVIE_019740 was used for the motif search. NVIE_001000 and NVIE_001020 are suggested to code for proteins involved with the cell surface while the rest are annotated as hypothetical proteins or proteins of unknown function.

The motif was also present in similar genes from *Candidatus* Nitrososphaera evergladensis and *Candidatus* Nitrososphaera gargensis, the two most closely related Thaumarchaeal species to *N. viennensis* (see Dataset S1 for a list of the identified genes and Fig. S6 for alignments of the motif across all three species). The conservation of this promoter in these other species suggests that a similar transcriptional pattern would be observed in both *Ca*. N. evergladensis and *Ca*. N. gargensis under Cu limitation.

The most strongly downregulated gene in Cu-limited cultures was a putative surface-associated Ca^2+^-binding protein (CBP_a, NVIE_001000; log_2_ FC −3.51) (Fig. 3a). Perhaps the cell remodels its surface to accumulate Cu in response to Cu limitation as previously reported [11, 57]. It is also possible that the Cu-limited cells inhibit cell aggregation by downregulating the putative surface-associated Ca^2+^ binding protein to enhance Cu uptake.

### Responses in energy metabolism and carbon fixation

**Fig. 5:**
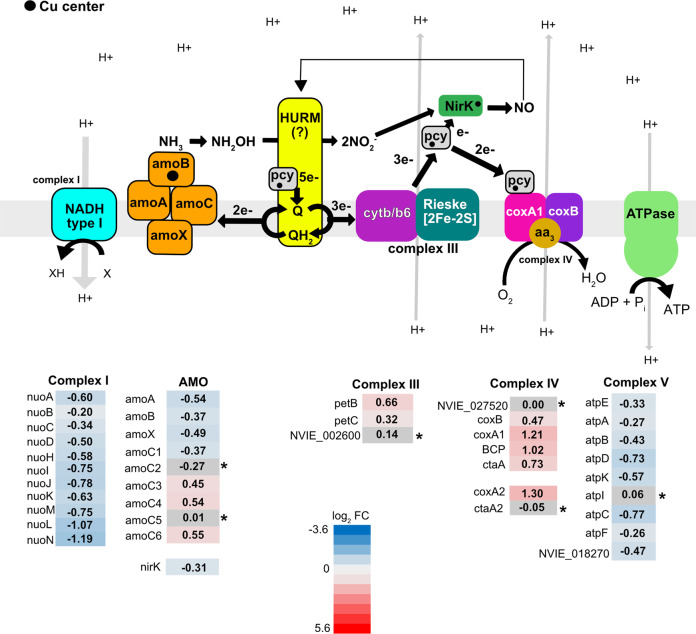
A schematic showing the expression of Cu and non-Cu containing genes potentially involved in the ammonia oxidation and electron transport pathways. Blue color is downregulated and red is upregulated; ● circle stands for Cu with an unknown redox state. A grey background with an asterix represents genes that were not differentially expressed. A *p* value < 0.01 cutoff was used to determine significance. (pcy) NVIE_002600 and NVIE_027550 (putative blue (type 1) copper domain containing proteins), NVIE_027520 cytochrome c oxidase subunit, NVIE_018270 putative ATPase subunit H, HURM; represents the hydroxylamine ubiquinone redox module [58] yet to be identified in AOA. NVIE gene information for all genes in this figure can be found in Dataset S1. Figure adapted from Qin et al. [26], Kerou et al. [9], and Kozlowski et al. [59].

In accordance with growth inhibition upon Cu limitation, most genes central for energy metabolism, i.e., the AMO complex, electron transport chain (ETC), and genes involved in carbon fixation, were downregulated. For the ETC, these included all subunits of complex I, most of the genes encoding for the archaeal ATP synthase (complex V), and genes potentially encoding plastocyanins [8]. It is hypothesized that plastocyanins form part of the archaeal HURM (hydroxylamine: ubiquinone redox module) and may shuttle electrons between protein complexes, similar to c-type cytochromes [58, 59]. Because of its ammonia-oxidizing metabolism, *N. viennensis* does not rely on complex I as a source of electrons for the ETC. Therefore, it has been suggested that complex I could be operating in reverse, possibly supplying a source of reducing equivalents to the cell for carbon fixation and biomass accumulation [58, 60, 61]. In this hypothesized model, complex I would consume H^+^ generated by the ETC and deplete the available proton motive force (pmf) (Fig. 5).

In contrast to complex I and complex V, other ETC components that contribute to pmf, (i.e., complex III (cytochrome b/b6 and Rieske [2Fe-2S]) and complex IV (*coxA1* and *coxB*)) were upregulated under Cu limitation (Fig. 5). A possible explanation for the ETC complexes being regulated in different ways is that the cell is attempting to conserve pmf, given that the pmf consuming complexes (complex I and complex V) were downregulated while the pmf contributing complexes (complex III and complex IV) were upregulated. A second possibility is that complex III and complex IV are upregulated to maintain the quinone and plastocyanin electron carriers in an oxidized state. The shift towards oxidized electron carriers could help to ensure that any electrons introduced into the ETC by the AMO complex are channeled directly towards the ETC because of the high supply of available electron acceptors.

Ammonia monooxygenase genes *amoA*, *amoB*, *amoX*, and *amoC1* were downregulated while AMO subunits *amoC3*, *amoC4*, and *amoC6* [9] were upregulated (Fig. 5). There are six copies of *amoC* in the genome [9] and it is unknown if all or only some interact with the AMO complex in *N. viennensis*. However, it was suggested that additional copies of *amoC* could ensure that the AMO enzyme is stable under conditions of stress [9] similar to findings in AOB [26, 62]. Although the cell seems to be reacting to energy limitation, AMO, the only source of energy, is downregulated. This is counter-intuitive but can be explained by limited Cu available to the cell, which is needed for this enzyme complex. Consequently, a transcriptional response that lowers the transcription of AMO is initiated. It should be noted that although transcription of AMO genes is lower under Cu-limited conditions compared to Cu-replete conditions, they still have some of the highest overall transcription levels in the cell (Dataset S1).

**Fig. 6:**
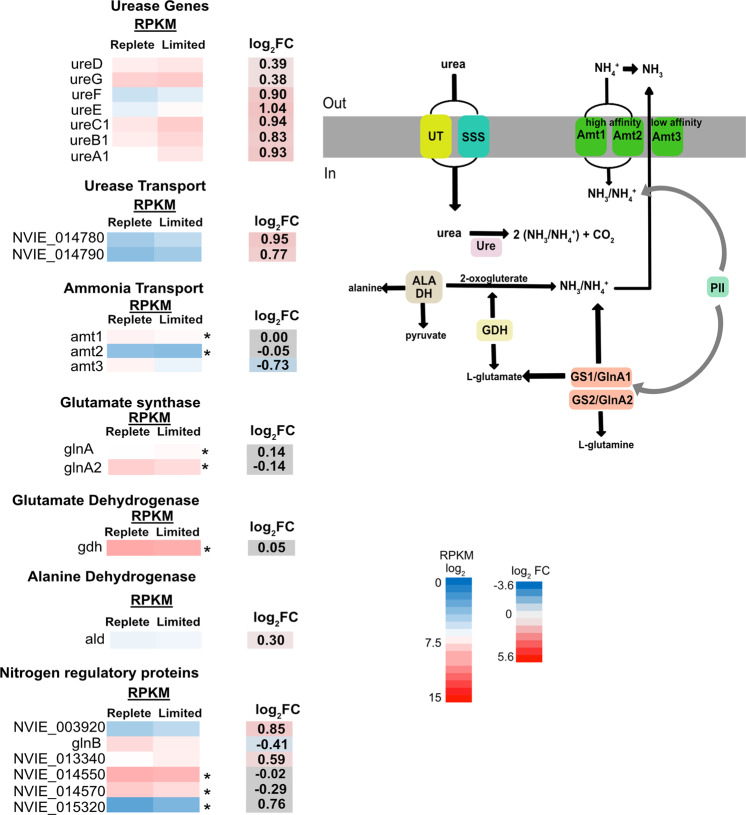
A schematic showing the expression of ammonia transporter, urease, and transcriptional regulator genes. For RPKM log_2_ and log_2_ FC, blue color is downregulated and red is upregulated. A grey background with an asterix represents genes that were not differentially expressed. A *p* value < 0.01 cutoff was considered as significantly expressed.

Genes involved in nitrogen uptake also showed distinct expression patterns. *N. viennensis* encodes three ammonia transporters, two urea transporters, and a urease operon (Fig. 6). Two of the ammonia transporters are thought to be high-affinity transporters and the other to be a low-affinity transporter [63]. The low-affinity transporter, *amt3* (NVIE_023840) (Fig. 6) was downregulated under Cu limitation while the presumed high-affinity transporters were unaffected. The differential regulation of ammonia transporters in response to environmental stimuli was also observed in *N. maritimus* [64]. Genes within the urease operon and urea transporters were all upregulated. The downregulation of the low-affinity transporter and the upregulation of the urease genes and urea transporters (even in the absence of urea) initially suggest a cellular response to ammonia limitation. This response regarding the low-affinity ammonia transporter was shown in previous studies of *N. viennensis* and other AOA when the cells were starved of ammonia [26, 63, 64]. However, in this study, the cells appear to be starved of energy, not ammonia. This is shown by the clear presence of ammonium in the medium at the time the cultures were harvested (Fig. 1). Therefore, it is likely that these cellular responses are regulated by an arrest of the ETC, rather than the physical presence or absence of ammonia.

As previously mentioned, the expression of the presumed high-affinity ammonia transporters was unaffected. If these importers responded to energy limitation, they would likely be upregulated similar to the urea transporters. This not being the case, it implies that their expression is regulated by a different system that could potentially sense the physical presence or absence of ammonia despite the energy-limited state of the cell. Moreover, the lack of differential expression in ammonia assimilation enzymes indicates that these proteins are unaffected by the low energy state of the cell and/or support the assumption that ammonia assimilation is not a limiting growth factor for the cell. A complex regulatory system involving nitrogen and energy should not be surprising as these two substrates are fundamentally intertwined in ammonia-oxidizing microorganisms.

Cu-limitation resulted in downregulation of genes encoding enzymes that consume NADPH (e.g., *asd*, *msr*) in the 3-hydroxypropionate/4-hydroxybutyrate (3HB/4HB) carbon fixation cycle (Fig. S9). Due to the low energy status of the cell, NADPH reducing equivalents, normally available to reduce inorganic carbon into organic carbon compounds, is likely scarce. As discussed above, one source of reducing equivalents could be the reverse complex I reaction (Fig. 5). However, reverse complex I would be unable to directly produce NAD(P)H due to the lack of *nuoE*, *nuoF*, and *nuoG* subunits in *N. viennensis* [9] and other archaea [65].

The non-oxidative pentose phosphate pathway (which produces precursors that can be converted to nucleotides [66]), ribosomal proteins, information processing genes, and most of the lipid biosynthesis pathway were also downregulated (Dataset S1). These observations match the functional enrichment of translation, replication, and nucleotide metabolism genes within the downregulated group (Fig. 2) and the general growth impairment of cells upon Cu limitation (Fig. 1).

### Different reactions to Cu limitation in N. viennensis and N. maritimus

**Fig. 7:**
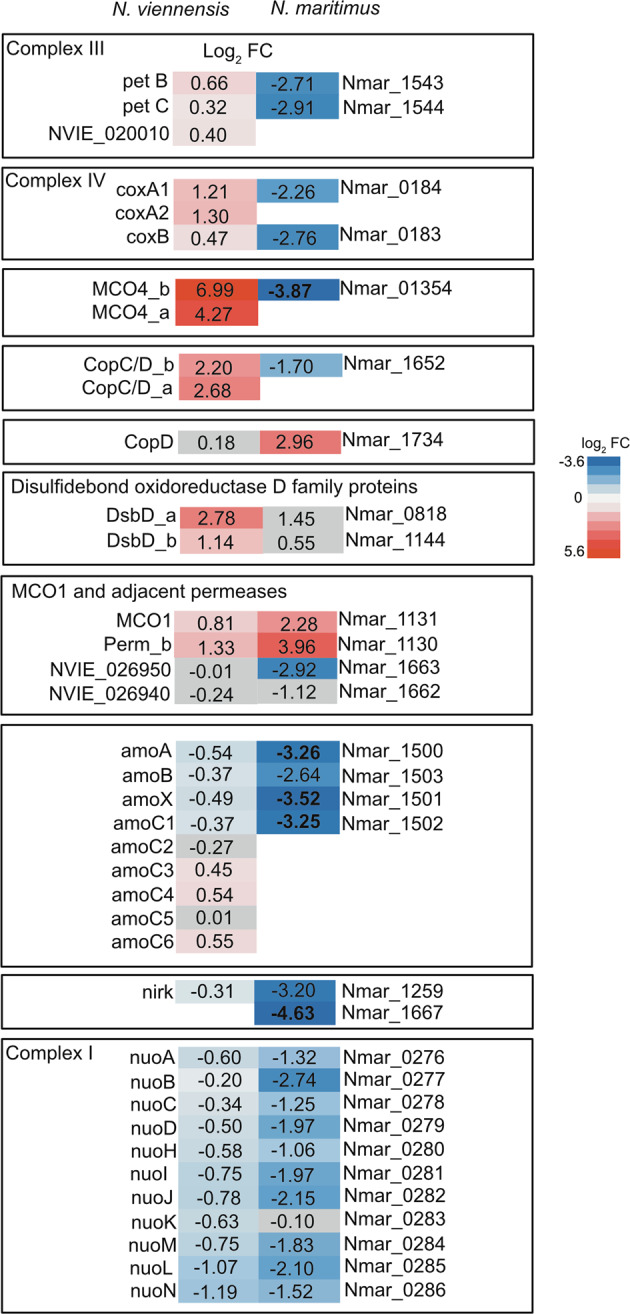
Comparison of selected genes from *N. viennensis* and *N. maritimus* under Cu limitation. Values represent the log_2_ fold change of genes compared to Cu-replete conditions A grey background represents genes that were not differentially expressed in *N. viennensis* (*p* value < 0.01) or *N. maritimus* (*p* value < 0.05) [26] respectively.

While *N. maritimus* showed similar overall responses to Cu limitation by downregulating general energy and carbon fixation pathways and slowing growth, Cu-acquisition or coping with Cu-limitation clearly differs from that in *N. viennensis* [26] (Fig. 7). Opposite reactions of complex III and complex IV in the ETC in response to the same environmental stimuli indicate a distinct regulation of energetic pathways between *N. viennensis* and *N. maritimus*, although the pathway itself is highly conserved among AOA. This distinction in regulatory patterns is also seen by the differing response in expression of homologous MCO4 [9] genes between the two organisms. Curiously, homologous MCO1 genes [9] show conflicting patterns in *N. maritimus*. In the case of MCO4, this would suggest that MCO4 plays a different physiological role in *N. maritimus*, possibly one that is not Cu related. In addition, each organism contains at least one CopC/D fusion protein and a lone CopD protein. Based on this comparison, *N. viennensis* relies on upregulation of the CopC/D fusion protein while *N. maritimus* rather upregulates *copD*. As CopD is a membrane bound transporter, *N. maritimus* likely utilizes a chaperone protein other than CopC to deliver Cu to CopD for transport into the cell. Alternatively, CopC/D could be linked to the proper assembly and function of complex IV as both are upregulated in *N. viennensis* and both are downregulated in *N. maritimus*. A final key difference is the upregulation of DsbD family proteins (arCOG02398) in *N. viennensis*. Perhaps, the upregulation of MCO4 and DsbD in *N. viennensis*, which are presumed to interact with Cu^+^, and the downregulation or lack of response respectively in *N. maritimus* is a reflection of the different availability of Cu^+^ in the very different environmental niches that these organisms occupy. Although Cu^2+^ is known as the bioavailable form in aerobic soil [67] and oxygenated sea-water environments [68], Cu^+^ is more likely to be found in soil environments that occasionally experience anoxic conditions and can possibly be stabilized at least to a certain extent by humic acids upon soil re-oxidation [69, 70]. Such environments include wetlands and riparian floodplain soils.

In neutral and alkaline soils, where organic and inorganic soil particles bind Cu^2+^ in soil solution and lower its bioavailability [71–73], these conditions could trigger the expression of CopC/D genes in soil organisms like *N. viennensis*. Another soil environment in which Cu concentrations are low and where expression of Cu-uptake genes could be triggered are heavily weathered and eroded soils. A study by Jenkins et al. [74] observed low *amoA* gene abundances of AOA in such soils with lowered total Cu concentrations.

Cu-C ratios and the types of ligands found in the soil environment differ from those in the marine environment [69]. In marine surface waters 10% of dissolved Cu has been shown to be Cu^+^ and in estuarine environments up to 80% of Cu could be Cu^+^ [75, 76]. Copper in these environments can be bound by thiol ligands, reduced sulfur species or colloidal ligands that lower its bioavailability [77–79]. In surface waters of Hood Canal, Washington, bioavailable Cu^2+^ and AOA *amoA* gene abundances were found to be low in surface waters [78]. Thus, *N. maritimus* inhabiting these environments might trigger the expression of genes encoding transporters that differ from those observed in this study to facilitate uptake of ligand-bound Cu.

## Conclusions

Results show that Cu-limitation in *N. viennensis* leads to high expression of several genes encoding MCOs and CopC/D proteins, which may be involved in Cu uptake. This study also puts forward the idea that Cu limitation triggers an energy conservation response in *N. viennensis*. This energy conservation response appears to downregulate the expression of genes involved in the consumption of the pmf, ammonia oxidation, and carbon fixation pathways while upregulating genes involved in ammonia acquisition by inducing the urease operon.

**Fig. 8:**
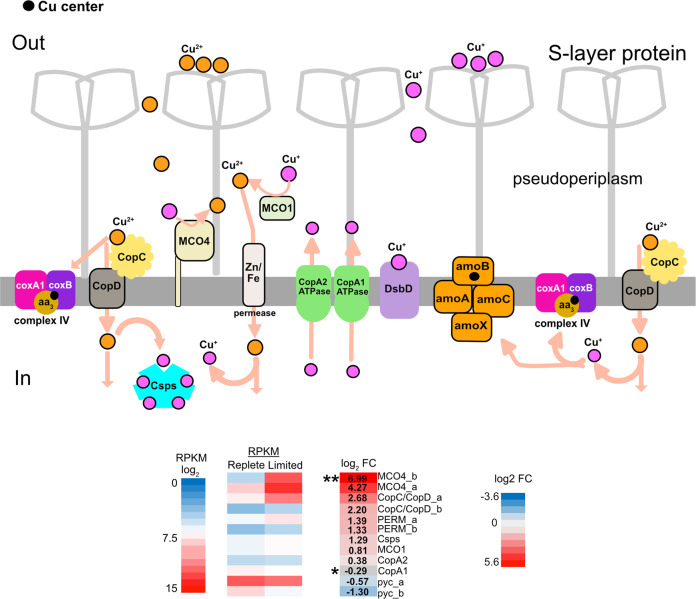
A schematic showing the expression of MCOs and Cop genes under Cu limiting conditions. ● circle stands for Cu with an unknown redox state, Cu^2+^, and Cu^+^. For RPKM log_2_ and log_2_ FC, blue color is downregulated and red is upregulated. (**) log_2_ fold change value is higher than pictured in the scale. A grey background with an asterix represents genes that were not differentially expressed. A *p* value < 0.01 was considered as significantly expressed.

In summary, Cu uptake could involve binding and uptake of Cu^2+^ by CopC/D fusion proteins (Fig. 8). CopC could also act as a chaperone helping to assemble cytochrome *aa*_*3*_ oxidase and AMO by transferring Cu^+^ to these proteins. MCO1 and MCO4 could bind and oxidize Cu^+^ to Cu^2+^, which could then be taken up either through a permease (encoded adjacent to MCO1) or an unknown divalent transporter. Once acquired, Csps could sequester Cu inside the cell. CopA1/A2 could function as Cu exporters and DsbD could possibly bind Cu^+^ and transfer it to chaperones in the periplasm for assembling Cu containing proteins or to import Cu into the cell.

Overall, this analysis proposes possible regulatory networks in terrestrial AOA, while highlighting distinct differences in adaptation strategies employed by different AOA clades in accordance to their ecophysiology.

## Supplementary information

Supplementary Information

Figure S1

Figure S2

Figure S3

Figure S4

Figure S5

Figure S6

Figure S7

Figure S8

Figure S9

Table S1

Table S2

Dataset S1
