# Supplementary material for: Genome wide transcriptomic analysis of the soil ammonia oxidizing archaeon Nitrososphaera viennensis upon exposure to copper limitation
Source: ISME J. 2020 Jul 14;14(11):2659–74. doi: 10.1038/s41396-020-0715-2 (PMC7785015; doi:10.1038/s41396-020-0715-2)
Supplement: Supplementary file 1 — Supplementary Information [file 41396_2020_715_MOESM1_ESM.docx]

**Supplementary Material**

**Genome wide transcriptomic analysis of the soil ammonia oxidizing archaeon *Nitrososphaera viennensis* upon exposure to copper limitation**

Carolina Reyes^[[1]](#footnote-1)^ (a)(b)(c)(#), Logan H. Hodgskiss^1^ (b)(c), Melina Kerou (b)(c), Thomas Pribasnig (b)(c), Sophie S. Abby (d), Barbara Bayer (c)(e)*, Stephan M. Kraemer (a)(c), Christa Schleper (b)(c)(#)

(a) University of Vienna, Centre for Microbiology and Environmental Systems Science, Department of Environmental Geosciences, Althanstrasse 14, UZA2, 1090 Vienna, Austria. (b) University of Vienna, Department of Functional and Evolutionary Ecology, Archaea Biology and Ecogenomics Unit, Althanstrasse 14, UZA1, 1090 Vienna, Austria. (c) University of Vienna, Environmental Science Research Network (ESRN), Faculty for Geosciences, Geography and Astronomy, Althanstrasse 14, UZA2, 1090 Vienna, Austria. (d) University Grenoble Alpes, CNRS, Grenoble INP, TIMC-IMAG, 38000 Grenoble, France. (e) University of Vienna, Department of Limnology and Oceanography, Division of Bio-oceanography, Althanstrasse 14, UZA1, 1090 Vienna, Austria.

*Present address: University of California Santa Barbara, Department of Ecology, Evolution and Marine Biology, 93106-9620 Santa Barbara, USA

#Corresponding Authors: Carolina Reyes ([creyes6@gmail.com](mailto:creyes6@gmail.com)) and Christa Schleper ([christa.schleper@univie.ac.at](mailto:christa.schleper@univie.ac.at))

Running Title: Transcriptome of Cu-limited *Nitrososphaera viennensis*

Keywords: AOA, nitrogen, ammonia oxidation, copper, archaea

**This article includes:**

**Supplementary figure legends**

**Supplementary dataset description**

**Supplementary methods**

**Supplementary references**

**Supplementary figures and legends S1-S9**

**Supplementary tables S1-S2**

**Figure Legends Supplementary Material**

**Figure S1.** Nitrite concentrations in batch cultures of *N.viennensis* grown in 30 mL polystyrene bottles under Cu-replete and Cu-limited (Cu10 and Cu12) conditions as a function of the calculated free Cu^2+^ species concentration. The data and error bars represent the average and standard deviation of multiple biological replicates (≥ 4) from several experiments.

**Figure S2**. Principal component analysis of rlog transformed expression data of Cu-limited (Lim) and Cu-replete (Rep) samples. The x-axis describes variability between Cu-limited and Cu-replete samples. The y-axis describes variability between the replicates of each condition. Ellipses represent concentration ellipses for each group at an ellipse.level=0.95.

**Figure S3**. Alignment of 2dMCOs showing the predicted Cu binding sites. 2dMCO sequences from the following microorganisms were aligned using the Clustal Omega multiple alignment web interface (<https://www.ebi.ac.uk/Tools/msa/clustalo/>) using default parameters: WP_084790903.1 copper oxidase; NVIE_019250 [*Nitrososphaera viennensis*], WP_075054901.1 copper oxidase; NVIE_017730 [*Nitrososphaera viennensis*], WP_075053492.1 copper oxidase; NVIE_000600 [Nitrososphaera viennensis], NP_711736.1 multicopper oxidase-like protein [*Leptospira interrogans serovar* Lai str. 56601], WP_011610637.1 multicopper oxidase [*Trichodesmium erythraeum*], WP_010998083.1 MULTISPECIES: multicopper oxidase [*Nostocaceae*], WP_011871074.1 copper oxidase [*Herminiimonas arsenicoxydans*], ADV66099.1 multicopper oxidase type 3 [*Deinococcus maricopensis* DSM 21211], EIA07124.1 multicopper oxidase [*Flavobacterium frigoris* PS1], ACA85706.1 multicopper oxidase type 3 [*Shewanella woodyi* ATCC 51908], KXK47905.1 multicopper oxidase [*Nitrosomonas europaea*], WP_013032143.1 multicopper oxidase [*Nitrosococcus halophilus*].

**Figure S4**. Alignment of the *N. viennensis* CopC/D amino acid sequences to CopC representative sequences from Lawton et al. (Biochemistry 55:2278-2290, 2016). Amino acid sequences were aligned using the Clustal Omega multiple alignment web interface (<https://www.ebi.ac.uk/Tools/msa/clustalo/>) using default parameters. Copper binding residues are boxed in black. Representative sequences are from the following organisms: *Nitrososphaera viennensis* (NVIE_014300, NVIE_014310), (NTE) *Nitrososphaera evergladensis* SR-1 (IMG Id 2586122348), (PG) *Pseudomonas* sp. GM17 (IMG ID 2503652063), (OB3b) *Methylosinus trichosoporium* OB3b (IMG ID 2507406428), (PR) *Pseudomonas fluorescens* R124 (IMG ID 2503652063), (MT) *Mycobacterium tusciae* JS617 (IMG ID 2508745664), and (SY) *Sphinogobium yanoikuyae* XLDN2-5 (IMG ID 2549030586).

**Figure S5**. Phylogeny and protein domain architecture of CopD proteins found in AOA (Nitrososphaeria, Thaumarchaeota). This phylogenetic tree was built on the basis of the alignment of the shared protein domain: CopD. The sequences were retrieved by sequence similarity search from a dataset representative of the diversity of the tree of life (see Supplementary Methods). The protein domain architecture is displayed for each sequence, as retrieved using PFAM domains' annotation. For AOA, the name of the orders is indicated along the tree with colored strips, whereas for other lineages, only the two highest taxonomic ranks are indicated (“phylum” and “kingdom” from left to right). Sequences found in *N. viennensis* are shown in red. DUF4149 (“Domain of Unknown Function”) domains that were overlapping CopD domains were ignored for display for two sequences: *Sphaerobacter thermophilus* DSM 20745 (WP_012872233.1) and *Candidatus* Nitrosocosmicus exaquare G61 (WP_148686115.1). “CopC” stands for protein domain PF04234.8 (“Copper resistance protein C”), “CopD” for protein domain PF05425.9 (“Copper resistance protein D”), “SGL” for PF08450.8 (“SMP-30/Gluconolaconase/LRE-like region”), “YtkA” for PF13115.2 (“YtkA-like”) and “FixH” for PF05751.7. Support values correspond to UltraFast bootstraps computed by IQ-Tree.

**Figure S6**. (A) Cop A1 and A2 (copper-exporting P-type ATPase A proteins), CopZ and CopT predicted gene clusters along with their gene expression information. A p-value cut-off of 0.01 was used to determine significance. Gene arrow colors represent log_2_ fold change of genes in copper limited cultures. Gray arrows are not differentially expressed. U = unknown protein, E = exported unknown protein. (B) Alignment of down-regulated genes with a motif sequence (5’-RATTTAAABATAGATTTAAATAATAAS-3’) from *N. viennensis* (NVIE) with other Thaumarchaeota genes containing a homologous motif. Ngar = *Nitrososphaera gargensis*, NTE = Candidatus Nitrososphaera evergladensis. Predicted translational start codons are boxed and text is in red. Locus tag information for all genes can be found in Dataset S1.

**Figure S7**. (A) *N. viennensis* CopA1 ATPase predicted metal binding sites. Amino acid metal binding sites information was gathered from several studies and reviews [1][2][3] [4]. N-MBS = N terminal metal binding site, TM-MBS = transmembrane metal binding site, C-domian = C terminal metal binding site, A-domain = actuator phosphatase P-domain = phosphorylation, N-domain = nucleotide binding. Arrow indicates path of Cu delivery by CopZ to TM-MBS (B) *N. viennensis* CopA2 predicted metal binding sites. Amino acid metal binding sites information was gathered from several studies and reviews cited above. TRASH= motif involved in metal coordination, N-MBS = N terminal metal binding site, TM-MBS = transmembrane metal binding site, C-domain = C terminal metal binding site, A-domain = actuator phosphatase, P-domain = phosphorylation, N-domain = nucleotide binding. Arrow indicates path of Cu delivery by CopZ to TM-MBS.

**Figure S8**. (A) Alignment of *N. viennensis* up-regulated genes suspected to be involved in Cu metabolism with a motif consensus sequence of 5’-TTCSGSMTTTGWACYANT-3’. Curved line indicates a break in the alignment. * indicates a reverse complement sequence and red letters indicates the motif repeats itself twice upstream or downstream of the motif. Predicted translational start codons are boxed and text is in red. Boxed green nucleotides indicate the location of a second motif and are included with the other motif regions at the bottom of the alignment. MCO, multicopper oxidase; BCP, protein of unknown function with C-terminal blue type (1) copper domain. (B) Alignment of top down-regulated genes from *N. viennensis* (NVIE) with a consensus motif sequence (underlined in gray). Predicted translational start codons are boxed and text is in red. CSP, cell surface associated Ca^2+^ binding domain protein, aemolysin type; CBP_a, putative surface associated Ca^2+^-binding protein; CWSP, putative cell wall surface anchor family protein; Lrp, transcriptional regulator Lrp family. Locus tag information for all genes can be found in Dataset S1.

**Figure S9**. A schematic of the carbon fixation and the tricarboxylic acid (TCA) pathways of *N. viennensis* showing the genes expressed in each pathway. FC in blue color is downregulated and red is upregulated. A p-value < 0.01 was considered as significantly expressed. Asterisk (*) symbol and a grey background indicates gene was not significantly expressed. Locus tag information for all genes can be found in Dataset S1.

**Dataset in Supplementary Material**

Dataset S1: Excel sheets with all up and downregulated genes at adjusted p-values of 0.01, excel sheets with genes used to find the identified motifs, excel sheet with list of homologous downregulated genes with the identified motifs, excel sheets listing gene locus information for genes in figures, excel sheets of ribosomal proteins, the non-oxidative pentose phosphate pathway, and genes encoding proteins containing Cu or Fe cofactors, excel sheet with list of species used for phylogenetic analysis of CopD, and excel sheet summarizing proteomic results.

**Supplementary Methods**

*Media Preparation*

Ultrapure water (18.2 MΏ cm, 2 ppb TOC) was used to prepare all media and to rinse plastic bottles. To reduce Cu contamination all media components were prepared in plastic bottles that had been washed with a mild detergent and then acid washed for several weeks, first in 3.8 % (v/v) HCl, and then in 7.4 % (v/v) nitric acid. Bottles were rinsed five times with ultrapure water in between acid washes and following the nitric acid wash. Preparation of all media components and handling of cultures was done in a trace metal free clean room environment with a HEPA filtration system. One liter of FWM medium [1 g NaCl, 0.4 g MgCl_2_·6H_2_O, 0.1 CaCl_2_·2H_2_O, 0.2 g KH_2_PO_4_, 0.5 g KCl], amended with 1 ml trace element solution [HCl 100 mM, H_3_BO_3_ 0.5 mM, MnCl_2_·4H_2_O, CoCl_2_ · 6H_2_O, NiCl_2_· 6H_2_O, ZnSO_4_ ·7H_2_O, Na_2_MoO_4_ ·2H_2_O, 0.01 mM CuCl_2_ · 2H_2_O], 1 ml vitamin solution [0.08 mM biotin; 0.05 mM folic acid, 0.48 mM pyrodoxin HCl, 0.14 mM thiamine HCl, 0.13 mM riboflavin, 0.4 mM nicotinic acid, 0.2 mM D-L-panthothenic acid, 0.36 mM p-aminobenozic acid, 1.4 mM choline chloride, 7.3 x 10^-6^ M vitamin B12], 7.5 µM ferric sodium EDTA, 2 mM NH_4_Cl, 2 mM NaHCO_3_ (as carbon source) and 1 mM sodium pyruvate (as a free radical scavenger), was used for growth. The medium was buffered with 10 mM HEPES/ 6 mM NaOH to pH 7.5. FWM, trace element mixture, vitamin solution, NH_4_Cl, NaHCO_3_, sodium pyruvate and HEPES buffer were filter sterilized with a sterile 0.2 µm PVDF filter and stored in the attached polystyrene bottles at 4°C.

*Culture Conditions*

The main culture of *N. viennensis* was initiated from a 0.5 mL 40 % glycerol stock by first spinning down the cells at 16,168 x g for 30 min at 4 °C, removing the supernatant, and re-suspending the cell pellet in 0.5 mL FWM. The resuspended cells were used to inoculate a 500 mL flask containing FWM medium supplemented with kanamycin (100 μg/mL) to prevent bacterial contamination. *N. viennensis* cultures were incubated at 42 °C, in the dark without shaking. Growth was monitored by following NH_4_^+^ consumption and NO_2_^-^ production using two modified colorimetric assays [5][6] described below. Once Cu-replete cultures had consumed ~ 1 mM NH_4_^+^, all cultures (including Cu-limited cultures) were split into subcultures by transferring cultures to 20 mL of FWM in polystyrene tubes supplemented with kanamycin (100 μg/mL) and carbenicillin (100 μg/mL) using a 0.25 % inoculation volume. To ensure a normal growth cycle, subcultures were continuously transferred for several weeks before PCR testing the cultures for bacterial contamination and initiating the Cu-limitation experiments.

*Nucleic Acid Extraction and PCR Testing*

Nucleic acids were extracted from 1 mL of cells in exponential growth phase. Cells were centrifuged at 16,168 x g (4 °C) for 30 minutes. The supernatant was discarded and cells were resuspended in SDS Extraction Buffer [0.14 M NaCl, 0.02 M Na_2_SO_3_, 0.1 M Tris/HCl, 0.5 M EDTA pH 8 and 1 % SDS]. This mixture was transferred to sterile 2 mL microtubes containing ~ 0.5 g of 0.1 mm zircona beads that had been baked at 180 °C for at least 3 hours. Phenol-chloroform-isoamyl alcohol (0.5 mL) [PCI, 25:24:1 (vol:vol:vol); pH 6.7-8] was first added to facilitate DNA recovery. Cells were lysed by bead-beating at 4 m/s for 30 s using a Fast Prep-24 instrument (MP Biomedicals). After bead beating and removal of PCI, chloroform-isoamyl-alcohol (0.5 mL) [CI, 24:1 (vol:vol)] was added to samples. During the PCI and CI extraction steps, tubes were centrifuged at 16,168 x g for 10 min at 4 °C. Between PCI and CI steps, before centrifugation, tubes were shaken heavily. Finally, nucleic acids were precipitated from the aqueous phase by adding 1 µL of glycogen and 2 volumes of 30 % polyethylene glycol (molecular weight 5000-7000) at 4 °C overnight, followed by centrifuging at 16,168 x g at 4 °C for 30 min. Pellets were washed with 1 mL of 4 °C cold 70 % ethanol, air dried for 3-5 min in a vacuum [Eppendorf Vacufuge Plus, 30 °C/AQ setting] and resuspended in 50 μL of DNase-free water. SDS Extraction Buffer, PEG and ethanol solutions were made using DNase-free water. Total DNA concentration was measured using the NanoDrop Spectrophotometer ND-1000 (PeqLab VWR International GmbH) following the manufacturer’s instructions.

DNA verification of the culture samples was assessed by performing a PCR using two different archaea primer sets including: 0.2 mM Arc109f [5’-ACKGCTCAGTAACACGT-3’] [7] and Arc 1492r [5’-GYYACCTTGTTACGACTT-3’] [8], CamoA-19f [5’-ATGGTCTGGYTWAGACG-3’] originally published by [9] and modified by [10], and TamoA-692r [5’-GCCATCCATCKRTANGTCCA-3’] [11] and possible bacterial contamination was assessed using 0.2 mM of primers 27f[5’-AGAGTTTGATCCTGGCTCAG-3’] and 1492r [5’-GGTTACCTTGTTACGACTT-3’][12]. 2 µL of DNA sample was used along with the following: 200 µM dNTP mix (Thermo Fisher), 0.002 mg/mL BSA, 0.15 µL, Dream-Taq DNA polymerase (Thermo Fisher), 1X Dream-Taq buffer and DNA free water in a 25 µl reaction volume. The following conditions were used for 16S rRNA primers: 95 °C for 5 min followed by 35 cycles of 94 °C for 30 s, 55 °C for 30 s and 72 °C for 2 min, and a final elongation step at 72 °C for 10 min. The following conditions were used for *amoA* primers: 95 °C for 5 min followed by 35 cycles of 94 °C for 30 s, 58 °C for 45 s and 72 °C for 45 s, and a final elongation step at 72 °C for 10 min. PCR reactions were checked on a 1 % low melting agarose gel.

*Cu-limited Cultures*

The trace element solution used in the Cu limited sub-cultures did not contain Cu. CuCl_2_·2H_2_O was supplemented to cultures at various free Cu and TETA concentrations as described in [13]. Briefly, the PhreeqC program [14] was used to estimate the Cu^2+^ and TETA concentrations needed to induce Cu limitation using the minteq.v4 database, stability constants from Anderegg et al. [15] Cu-limitation was assessed as a decrease in NH_4_^+^ consumption and NO_2_^-^ production relative to the Cu-replete culture. Total concentration of Cu in the FWM with additives was measured using inductively coupled plasma mass spectrometry (ICP-MS; Agilent 7700 instrument)[16].

*Ammonium and Nitrite Measurements*

Ammonium concentrations were determined based on a modified method [5]. Briefly 200 µL of sample was combined with 400 µL of FWM. 300 µL of Color Reagent [5.18 mM sodium salicylate, 2.15 x 10^-5^ M sodium nitroprusside, 0.1 M NaOH] was added next followed by 120 µL Oxidation Solution [3.91 x 10^-5^ M of dichloroisocyanuric acid]. After mixing, samples were stored in the dark at room temperature for 30 min. The absorbance of 200 µL samples were measured in a 96-well plate with a UV spectrophotometer [TECAN Sunrise Spectrophotometer] at 660 nm wavelength. NO_2_^-^ concentrations were determined by using a modified method [6]. Briefly, 20 µL sample was combined with 780 µL FWM and to this 200 µL of sulfanilamide/NED Reagent [0.058 M sulfanilamide, 1.9 mM N-(1-Napthyl)-ethylendiamin dihydrochloride, 2.22 M ortho-phosphoric acid 85 %] was added. After mixing, the samples were stored in the dark at room temperature for 30 min before measuring the absorbance at 545 nm wavelength.

*RNA Extraction*

For RNA extraction, 1.25 mL of an exponential phase 20 mL culture was used to inoculate a 1 L polystyrene bottle containing 500 mL of FWM. Cultures were harvested on different days as described below (Fig. 1) and total RNA was extracted from five biological replicates. Growth continued to be monitored in the remaining cultures until stationary phase. A sterile 60 mL syringe that was equipped with a 25 mm Swinnex filter holder with gasket with o-ring (Millipore) was used to withdraw culture. In this way, 250 mL (for RNA-Seq experiments) of cells were collected on a 25 mm 0.2 μm mixed cellulose ester filter membrane (Millipore). The filters were added to water treated with 1 % DEPC overnight and immediately autoclaved. Wet filters were used within 1-2 days. Following the filtration step, filters were inserted into 5 mL lysis tubes and frozen on dry ice. After thawing the filters, the extraction procedure was started by first heating the filter to 65 °C for 10 min. RNA was extracted from cells using the RNeasy Porewater extraction kit (Qiagen) according to the manufacturer’s instructions with a few exceptions. Samples were split into 500 μL sub-samples prior to addition of PWR 3 and 4 solutions. Following addition of PWR 3 and 4, multiple loads for each sample were added to the spin filter. The on-column DNase I digest was omitted. Nucleic acid was eluted in 100 μL of RNase free water.

*DNase I Digest and Total RNA Preparation for Sequencing*

Nucleic acid was treated with RQ1 DNase I (Promega) using the manufacturer’s instructions. DNase I and any remaining inhibitors were removed from the RNA with Zymo One Step PCR Inhibitor removal kit following the manufacturer’s instructions. The DNA High Sensitivity Bioanalyzer Chip (Agilent) was used, following the manufacturer’s instructions, to exclude the presence of genomic DNA. The concentration of RNA extracted was estimated using the RNA Qubit HS (Thermo Fisher) assay and the integrity of the RNA was determined with the Agilent RNA 600 Nano Kit.

*Reverse Transcription (RT) PCR and Quantitative PCR (qPCR)*

The same samples that were sequenced were used in the RT-qPCR analyses. Prior to the RT step, 0.5-1 µg of total RNA was treated with Turbo DNase I (Thermo Fisher) following the manufacturer’s instructions. After checking for DNA contamination using MCO primers in PCR as described below, total RNA was reverse transcribed using the Protoscript II M-MuLV reverse transcriptase (New England Biosciences) following the manufacturer’s instructions. Reactions were cleaned up with the Monarch DNA cleanup kit (New England BioLabs) following the protocol for cDNA. As a normalization step, the concentration of cDNA was estimated using the Qubit ssDNA quantitation kit and all samples were diluted to 5 ng/μL prior to the qPCR step.

The Luna® Universal qPCR Master Mix (NEB) and the Mastercycler EP Gradient S (Eppendorf) qPCR thermocycler were used for absolute quantification of gene copy numbers. For each biological replicate sample or standard template, three technical qPCR replicates were tested. The primers listed in Table S2 were used to amplify the permease gene *perm_b* (NVIE_000590), the multicopper oxidase genes *mco1* (NVIE_000600) and *mco4_b* (NVIE_019250), and a putative surface associated Ca^2+^ binding protein encoding gene *cbp_a* (NVIE_001000). Expression of the 16S rRNA gene and *rpoB* (NVIE_013510) was also assessed for comparative purposes.

Standard templates were prepared by amplifying *mco1* (NVIE_000600), *perm_b*, (NVIE_001000), 16S rRNA and *rpoB* genes from DNA extracts of 5 Cu-replete and 5 Cu-limited cell samples of *N. viennensis,* using the primers described above. The following PCR conditions were used for all primers: 98 °C for 3 min followed by 30 cycles of 98 °C for 10 s, 60 °C for 20 s and 72 °C for 30 s, followed by a final elongation step at 72 °C for 4 min. PCR products were cleaned up with the Monarch DNA cleanup kit (New England BioLabs) following the protocol for PCR products and cloned into the pJET/blunt plasmid using the CloneJet PCR Cloning Kit (Thermo Fisher) according to the manufacturer’s instructions. Purified plasmids were linearized using Not I HF (NEB) restriction enzyme. Standard curves were generated by 10X serial dilutions of linearized plasmid stocks with a concentration of 0.2 x 10^8^ copies/μL (*perm_b* and *mco1* plasmids) and 0.2 x 10^9^ copies/μL for *mco4_b*, *cbp_a*, 16S rRNA, and *rpoB*. Final standards ranged from 10^1^ to 10^8^ copies of the targeted gene. Each 20 μL reaction mixture was prepared following the Luna Master Mix instructions. The following qPCR cycling conditions were used for all primers: 95 °C for 2 min followed by 40 cycles of 95 °C for 15 s and 60°C for 1 min. The specificity of each qPCR reaction was evaluated via melting curve. Relative difference in expression of genes *mco1*, *mco4_b*, *perm_b*, and *cbp_a* was first assessed by calculating the ∆Ct of each gene against the ∆Ct of 16S rRNA or *rpoB* (used as stably expressed reference genes). These values were then normalized to the average ∆Ct of 16S rRNA or *rpoB* genes from the Cu-replete cultures [17]. Following normalization, one Cu-replete sample (R3) was left out of further analyses as amplification of genes for this sample was one order of magnitude lower compared to other samples.

*Sequencing Analysis*

Raw reads were downloaded from the Vienna Biocenter Facitlity website and checked using md5sum. FastQC [18] version 0.11.5 was used to initially analyze sequences and the samtools package version 1.9 [19] (using htslib 1.9) was used to convert file types. Trimmomatic [20] version 0.36 trimmed adapter sequences and cropped reads using the options: SLIDINGWINDOW:4:15 LEADING:3 TRAILING:3 MINLEN:38 HEADCROP:13. The stand-alone program PrinSeq Lite [21] version 0.20.4 was used to remove sequences < 37 bp and/or with quality scores < 30. SortMeRNA[22] (version 2.1) separated rRNA sequences from total RNA using a curated reference database only containing *N. viennensis* rRNA sequences. Next, [23] “Bowtie2” [24] version 2.3.3.1 was used to align the remaining RNA sequences to the *N. viennensis* reference genome. The “featureCounts” [25] program, version 1.6.2, from the subread package assigned mapped sequencing reads to genomic features. While using featurecounts, a General Feature Format (GFF) annotation file with annotations from GenBank for *N. viennensis* from the National Center for Biotechnology Information (NCBI) website (<ftp://ftp.ncbi.nlm.nih.gov/genomes/all/GCA/000/698/785/GCA_000698785.1_ASM69878v1>) was used as a reference. Feature type was specified as “gene”, and attribute type was specified as “ID”. Classified ambiguous sequences were omitted from the final output prior to further processing. The count matrix from feature counts was used as input for R version 3.2 [26] with RStudio version 0.99.902 [27]. The R bio-conductor package “DESeq2” version 1.10.1 [28] was used to normalize reads and to perform differential expression analysis. In order to lower the false discovery rate (FDR), the default p-value adjustment in DESeq2 was used (Benjamini-Hochberg method) to determine relevant p-values (p-adj in DESeq2 output). For principal component analysis, count data was normalized using the rlog function in DESeq2. A PCA plot was then produced from the normalized data using the R packages ggplot2 [29], ggrepel [30], and factoextra [31]. R scripts for the differential expression analysis and PCA plot production are found on Github (<https://github.com/hodgskiss/Nviennensis_copper_limitation>). Up and downregulated genes were assigned to functional categories based on their archaeal clusters of orthologous group (arCOG) categories [32]. A statistical hypergeometric test was performed (described below) to determine which categories were significantly enriched (p-value < 0.05) in the up or downregulated gene groups.

*arCOG Functional Group Assignment and Enrichment Analysis*

Archaeal Clusters of Orthologous Groups, also known as arCOGs [32] (2014 version), were assigned to genes of *Nitrososphaera viennensis* EN76, *Nitrosopumulis maritimus* SCMI, *Candidatus* Nitrososphaera gargensis Ga9.2, *Candidatus* Nitrososphaera evergladensis SR1, *Nitrosotenuis chungbukensis* MY2, *Nitrosoarchaeum limnia* SFB1, and *Candidatus* Nitrosopumulis sediminis AR2 using the COGnitor scripts, with an e-value threshold set to 10^-10^. Manual inspection of the protein families resulted in grouping into custom functional categories. Functional enrichment analysis was performed using an arCOG enrichment R script that utilizes the phyper() R function as previously described [33] ([https://github.com/amyschmid/histone_arCOG](https://github.com/amyschmid/histone_arCOG)). In brief, a hypergeometric distribution is used to determine the statistical chance of certain categories (in this case arCOGs) being drawn from a set (in this case the total arCOGs found in the genome).

*[RPKM Calculation](https://github.com/amyschmid/histone_arCOG)*

The resulting data from the featurecounts program was used to calculate the reads per kilo base per million (RPKM) for each gene in each sample. The counts for each sample were totaled and divided by one million. This factor was applied respectively for every gene in each sample (i.e. [number of counts for a gene]/factor). Each gene count was also divided by the length of the respective gene in kilobases. This final value, or RPKM, was log transformed with a base of two. Averages for each gene were taken for the copper replete and copper limited conditions and can be found in Dataset S1 for each gene.

*[Phylogenetic Analysis of the CopD Family in Thaumarchaeota](https://github.com/amyschmid/histone_arCOG)*

The sequences from the three proteins harboring CopD domains in *N. viennensis* (NVIE_013130 – WP_084790666.1, NVIE_014300 – WP_075054630.1 and NVIE_014310 – WP_075054632.1) were used to perform a blast similarity search against a database comprising a selection of genome sequences from 39 AOA, plus 341 genomes from representative clades of bacteria, archaea and eukaryotes (Dataset S1). Sequences corresponding to hits with an e-value lower than 10^-6^ were collected, and these hits were then screened with HMMER v3.1b2 [34] against PFAM v28.0 [35] for CopD domains. Sequences harboring CopD domains were selected, and the coordinates (“envelope”) of the CopD domains were used to extract parts of the sequences corresponding to these domains. CopD domains from selected sequences were thus aligned using the MAFFT program v7.310 (“linsi”) [36], resulting in a 175-sites alignment. A phylogenetic tree was built from this alignment using IQ-Tree v1.6.11 [37], with the best model selected (“-m TESTNEW”) and with the estimation of the support values (“-bb 1000 -alrt 1000 -safe”). The resulting tree was then annotated in iTOL [38] with the protein domain architecture as obtained with the PFAM screen. The figure (Fig. S5) was finalized using the InkScape program (<http://www.inkscape.org/>).

*Motif Analyses*

The web version of Multiple Em for Motif Elicitation (MEME) software Suite 5.0.5 was used to identify motifs in upstream regions of selected genes. For each analyzed upstream sequence, the first 100 bases upstream of the annotated translational start codon were considered unless the end of an upstream gene caused this region to be shorter. Upstream bases were extracted using The Artemis Software [39]. For genes in a presumed operon, the upstream start region of the operon was considered instead. Genes were presumed to be in an operon if the distance between the end of an open reading frame and the start of the next, on the same strand, was ~ < 40 bp. The option “any number of occurrences” was used to find repeated motifs for the selected up and downregulated gene groups. For the list of homologous genes in Thaumarchaeota, the search was limited to “search given strand only” within the advanced options. Once a motif was found in selected upregulated genes, it was used to screen against the top 25 upregulated genes using the web version of Find Individual Motif Occurences (FIMO) version 5.1.1 [40].

*Genes Neighboring Downregulated Genes and Homolog Search in Closely Related Thaumarchaeal Species*

Based on the observation that several downregulated genes containing the motif are located in the same genomic region, the motif search was adapted to contain not only the previously identified genes with the motif, but also NVIE_000950, NVIE_000960, NVIE_000970 and NVIE_000980, as most of these genes were also downregulated in the Cu-limited condition and are located between NVIE_000940 and NVIE_001000. The motif was found in the upstream region of NVIE_000970 and in the upstream region on the opposite strand in reference to the gene orientation for genes NVIE_000950 and NVIE_000980. Interestingly, this promoter region is largely palindromic resulting in NVIE_000990 and NVIE_000980 sharing the same conserved region. Although not confirmed in this study, it seems possible that bi-directional transcription, defined as transcription in opposite directions [41] from one core promoter, may occur.

While most of these additional genes do not have an annotated function, NVIE_000950 stands out as having a helix-turn-helix (HTH) ArsR and 4-vinyl reductase (V4R) signature. Whereas the V4R signature indicates a potential to bind hydrocarbons [42] the HTH ArsR domain corresponds to a group of transcriptional regulators known to respond to the presence of metals. In particular, they are known as repressors that bind to DNA in the absence of metals. In the presence of metals, these repressors are released from the DNA and transcription can proceed [43]. These types of regulators are also known to be involved in Cu regulation systems [44]. It is possible that such a repressor that binds to DNA regulates the downregulated genes identified here. Under Cu replete conditions, the binding of Cu to the repressor would release it from the DNA and allow transcription to proceed. NVIE_000950, or even the upregulated NVIE_014280, could be fulfilling this regulatory function leading to the downregulation of this gene cluster as both are detected in the proteome of copper limited cultures. However, this hypothesis would need to be verified by molecular methods. It should be noted that although NVIE_000950 was found in the proteome of copper limited cultures, it is not differentially expressed between the two conditions based on the p-value cutoff of 0.01.

To further investigate the occurrence of this sequence, the basic local alignment tool (BLAST) [45][46] was used to search for this promoter sequence in other areas of the *N. viennesis* genome. This resulted in a sixth gene being identified with the motif: NVIE_005310, a transcriptional regulator from the Lrp family. This gene, however, is not differentially expressed between the two conditions based on the p-value cutoff of 0.01.

With the exception of NVIE_000950, the identified promoter was found at a similar distance from the start codon of its respective gene. Because of the high level of conservation in nucleotide composition of this motif and its distance from the start codon, similar proteins to genes with this motif and/or in this cluster were searched for in other Thaumarchaeota species to determine if this motif sequence was more widespread. Similar proteins were identified by BLAST [45][46] results in the above mentioned Thaumarchaeota species (see “*arCOG Functional Group Assignment and Enrichment Analysis*” section for a list of species) or identified based on similarity and synteny using the MicroScope web interface Microbial Genome Annotation & and Analysis Platform (MAGE) [47]. Similar proteins for NVIE_000960 were not searched for as the motif was not as strong in the promoter region of this gene (Fig. S6B). A complete list of similar proteins can be found in Dataset S1. Upstream regions were taken for each of the identified genes and MEME was used to look for the motif sequence with the search limited to “search given strand only” within the advanced options. An alignment of promoter regions for genes with the identified motif can be seen in Fig. S6B.

*Combined Protein and RNA Extraction*

Proteins and RNA were extracted using a combined TRIzol (Invitrogen) extraction method from biomass frozen on filters stored at -80 °C from cultures L7 and L8. These methods closely follow those of Ott *et al.* (2019) [48] with the exception of combining RNA and protein extraction, rather than metabolites and proteins, in the same step. Additional slight changes were made in the protocol.

Filters were taken from the freezer and added to 2 mL homogenization tubes (Sarstedt, order number: 72.693.465) containing approximately 0.6 grams of Lysing Matrix B (MP Biomedical). 1 mL of TRIzol reagent was added to each tube and samples were then homogenized using a FastPrep-24 homogenizer (MP Biomedical) set at 4 m/s for 30s. After homogenization, samples incubated at room temperature for 15 min. Following incubation, 200 µL of chloroform was added and each sample was inverted five times before incubating at room temperature for another three minutes. Samples were then centrifuged at 16,168 x g and 4 °C to separate phases. After centrifugation, the lower phase (chloroform), containing proteins, and the upper phase, containing RNA, were removed into separate low bind 2 mL Protein LoBind tubes (Eppendorf).

To finish the protein extraction, 550 µL (approximately a 1:1 mix) of filtered water (MilliporeSigma Milli-Q Reference A+ System) was added to each protein sample. Tubes were inverted five times and incubated at room temperature for 3 min. Each sample was then centrifuged at 16,168 x g and 4 °C for 2 min to separate phases. The lower apolar phase containing proteins was transferred to a new 2 mL low bind epi. 1.5 mL of 0.1 M NH_4_Cl dissolved in methanol with 0.5 % β-mercaptoethanol was added to the apolar phase. Samples sat on ice for 3-4 hours before being stored overnight at -20 °C to allow proteins to precipitate. The next day, samples were centrifuged at 16,168 x g for 15 min at 4 °C to allow proteins to pellet. The supernatant was discarded and proteins were resuspended in 1.8 mL of ice cold methanol. The sample was sonicated in a Transsonic 700/H water bath (Elma) until proteins were completely resuspended. The samples were centrifuged at 16,168 x g for 10 min at 4°C and the supernatant was discarded. The washing process with methanol was repeated a second time. After the second methanol washing step, proteins were suspended in 1.8 mL of ice cold acetone. Again, each sample was sonicated in a Transsonic 700/H water bath (Elma) until proteins were completely resuspended. Each sample was centrifuged at 16,168 x g for 15 min at 4°C. The acetone supernatant was discarded and samples were allowed to air dry under a fume hood for 5-10 min (or until just after being fully dried) and then stored at -80 °C.

To finish RNA extraction, 10 µg of glycogen and 0.5 mL of isopropanol was added to each RNA sample (the previously separated aqueous phase). Each sample was allowed to sit for approximately 60 min at room temperature before being centrifuged at 12,000 x g for 10 min at 4°C. The supernatant was discarded and the RNA was resuspended in 1 ml of 75 % ethanol. Each sample was briefly vortexed and then centrifuged at 7,500 x g for 5 min at 4 °C. The supernatant was discarded and the RNA pellet was allowed to air dry. The dried pellet was resuspended in 25 µL of diethyl pyrocarbonate (DPEC) treated water and stored at -80 °C for later RT-qPCR analysis.

*Protein Digestion*

Extracted proteins were resuspended in 500 µL of extraction buffer (8 M urea in 50 mM HEPES buffer, pH 7.8) and allowed to shake for 30 min at 4 °C at 900 rpm. Protein concentration was then measured using the Bradford assay (Bio-Rad Cat. No. 500-0006) using a pre-made standard curve with bovine serum albumin (BSA) and an absorbance of 545 nm. Based on the results of the Bradford assay, a volume corresponding to 25 µg of protein was removed to a new low bind epi for each sample respectively. Extraction buffer was used to bring the total volume of each sample to 220 µL. 1,4-dithiothreitol (DTT) was added to get a concentration of 5 mM in each sample followed by incubation for 45 min at 37 °C and 700 rpm. Iodoacetamide (IAA) was then added to a concentration of 10 mM followed by incubation in the dark for 60 min at 30 °C and 700 rpm. A final addition of DTT was added to bring the total concentration up to 10 mM followed by incubation in the dark at room temperature for 15 min. To digest proteins, 5 µL of 0.1 µg/µL of mass spec grade rLysC (Promega) was added to each sample. Proteins were digested for 3 hours at 37 °C and 700 rpm. After the rLysC digestion, 660 µL of trypsin buffer (50 mM NH_4_HCO_3_, 2 mM CaCl_2_, 5 mM DTT, 10 % acetonitrile) was added to each sample. This is necessary to dilute the urea concentration to 2 M for the trypsin digestion. 1 µL of trypsin beads from a Poroszyme Immobilized Trypsin Cartridge (Applied Biosystems) were added to each sample. Trypsin digestion was carried out for 16 hours at 30 °C on a rotator. Following digestion, peptides were desalted using OMIX C18 pipette tips (Agilent Technologies). For this, samples were centrifuged briefly to pellet the trypsin beads before desalting. Tips were activated by washing with 100 µL of methanol and then washed twice with 100 µL of 0.1 % formic acid (FA). Peptide solution was then acidified with the addition of FA to a final concentration of 3 %. The solution containing peptides was pipetted through the C18 tips and then washed twice with 100 µL of 0.1 % FA. Peptides were then eluted and saved from the C18 tips by being washed twice with 100 µL of methanol. After desalting, peptides were dried in a ScanSpeed 40 speed vacuum with a ScanVac vacuum control and stored at -20 °C.

*Mass Spectrometry of Peptides*

To prepare the peptides for mass spectrometry analysis, peptides were resuspended in 250 µL of 2 % acetonitrile (ACN) and 0.1 % formic acid (FA). 5 µL were then injected into an EASY-Spray C18 column (2µm, 100Å 75 µm x 50 µm, Thermo). Peptides were eluted from the column for 150 min using a 90 min linear gradient starting from 96 % solvent A (0.1 % FA) and 4 % solvent B (80 % ACN, 0.1 % FA) to 35 % of solvent B with a flow rate of 0.3 µL/min. The linear gradient was followed by an increase of solvent B to 90 % over 1 min and held at 90 % for 8 min. Solvent B was then adjusted from 90 % to 4 % over 1 min and held at 4 % for the remaining 50 min. Ion source was an EASY-Spray source with a spray voltage of 1.9 kV. Mass spectrometry measurements were taken using an LC-QExactive-Plus (Thermo) with the following settings: 0 to 150 min; MS1: positive polarity, full scan range 380-1800 *m/z*, resolution 70,000, collision-induced dissociation (CID) fragmentation for the 20 most intense ions; MS2: loop count 20, resolution 17,500, scan range 200-2000 *m/z.*

*Peptide Analysis*

Output files were analyzed using MaxQuant version 1.6.3.3 [49] with a reference proteome for *N. viennensis* downloaded from the Uniprot website (proteome UP000027093). The variable modifications oxidation (M) and acetyl (Protein N-term) were allowed along with the fixed modification carbamidomethyl (C). Digestion mode was set for trypsin and lysC with a maximum missed cleavages of 2. The protein false discovery rate was set at 0.01 and a minimum number of peptides at 1. The mqpar.xml file with relevant parameters is available on Github (<https://github.com/hodgskiss/Nviennensis_copper_limitation>).

**References**

1. Raimunda D, González-Guerrero M, Leeber BW, Argüello JM, Argüello JM. The transport mechanism of bacterial Cu+-ATPases: distinct efflux rates adapted to different function. *Biometals* 2011; **24**: 467–75.

2. Solioz M, Odermatt A. Copper and silver transport by CopB-ATPase in membrane vesicles of Enterococcus hirae. *J Biol Chem* 1995; **270**: 9217–21.

3. Orell A, Navarro CA, Arancibia R, Mobarec JC, Jerez CA. Life in blue: Copper resistance mechanisms of bacteria and Archaea used in industrial biomining of minerals. *Biotechnol Adv* . 2010. , **28**: 839–848

4. Zoltner M. Structural and functional characterization of prokaryotic CPx-ATPases: evidence for a novel cobalt P-type ATPase. *PhD Thesis* . 2006. International School Bioscience Ruhr University, Bochum.

5. Kandeler E, Gerber H. Short-term assay of soil urease activity using colorimetric determination of ammonium. *Biol Fertil Soils* 1988; **6**: 68–72.

6. Griess P. Bemerkungen zu der Abhandlung der HH. Weselsky und Benedikt „Ueber einige Azoverbindungen”. *Berichte der Dtsch Chem Gesellschaft* 1879; **12**: 426–428.

7. Grosskopf R, Janssen PH, Liesack W. Diversity and structure of the methanogenic community in anoxic rice paddy soil microcosms as examined by cultivation and direct 16S rRNA gene sequence retrieval. *Appl Environ Microbiol* 1998; **64**: 960–9.

8. Delong EF. Archaea in coastal marine environments. *Proc Natl Acad Sci USA* 1992; **89**: 5685–5689.

9. Tourna M, Stieglmeier M, Spang A, Könneke M, Schintlmeister A, Urich T, et al. *Nitrososphaera viennensis*, an ammonia oxidizing archaeon from soil. *Proc Natl Acad Sci U S A* 2011; **108**: 8420–8425.

10. Pester M, Rattei T, Flechl S, Gröngröft A, Richter A, Overmann J, et al. amoA-based consensus phylogeny of ammonia-oxidizing archaea and deep sequencing of amoA genes from soils of four different geographic regions. *Environ Microbiol* 2012; **14**: 525–539.

11. Arce MI, von Schiller D, Bengtsson MM, Hinze C, Jung H, Alves RJE, et al. Drying and rainfall shape the structure and functioning of nitrifying microbial communities in riverbed sediments. *Front Microbiol* 2018; **9**: 2794.

12. Eden P a, Schmidt TM, Blakemore RP, Pace NR. Phylogenetic analysis of *Aquaspirillum magnetotacticum* using polymerase chain reaction-amplified 16S rRNA-specific DNA. *Int J Syst Bacteriol* 1991; **41**: 324–325.

13. Reyes C, Hodgskiss L, Baars O, Kerou M, Bayer B, Schleper C, et al. Copper limiting threshold in the terrestrial ammonia oxidizing archaeon *Nitrososphaera viennensis*. *Res Microbiol* 2020; 1–9.

14. Parkhurst DL. User’s guide to PHREEQC, a computer program for speciation, reaction-path, advective-transport, and inverse geochemical calculations. *Water-Resources Investig Rep* 1995.

15. Anderegg, Giorgio, Arnaud-Neu, Francoise, Delgado Rita, Felcman, Judith, Popov K. Critical evaluation of stability constants of metal complexes of complexones for biomedical environmental applications. *Pure Applied Chemistry* . 2005.

16. Technologies A. Agilent ICP-MS ( 8800 / 7700 ) Introduction System ( ISIS ). 2012. Agilent Technologies.

17. Pfaffl MW. A new mathematical model for relative quantification in real-time RT-PCR. *Nucleic Acids Res* 2001; **29**: 45e – 45.

18. Andrews S. FastQC: a quality control tool for high throughput sequence data. 2010.

19. Li H, Handsaker B, Wysoker A, Fennell T, Ruan J, Homer N, et al. The Sequence Alignment/Map format and SAMtools. *Bioinformatics* 2009; **25**: 2078–2079.

20. Bolger AM, Lohse M, Usadel B. Trimmomatic: a flexible trimmer for Illumina sequence data. *Bioinformatics* 2014; **30**: 2114–2120.

21. Schmieder R, Edwards R. Quality control and preprocessing of metagenomic datasets. *Bioinformatics* 2011; **27**: 863–864.

22. Kopylova E, Noé L, Touzet H. SortMeRNA: fast and accurate filtering of ribosomal RNAs in metatranscriptomic data. *Bioinformatics* 2012; **28**: 3211–3217.

23. Team RC. A language an environment for statistical computing. R Foundation for Statistical Computing, Vienna. 2013.

24. Langmead B, Salzberg SL. Fast gapped-read alignment with Bowtie 2. *Nat Methods* 2012; **9**: 357–9.

25. Liao Y, Smyth GK, Shi W. featureCounts: an efficient general purpose program for assigning sequence reads to genomic features. *Bioinformatics* 2014; **30**: 923–930.

26. R Foundation for Statistical Computing. A language and environment for statistical computing. 2016. Vienna, Austria.

27. Team Rs. RStudio: Integrated Development for R. 2016. Boston, MA.

28. Love MI, Huber W, Anders S. Moderated estimation of fold change and dispersion for RNA-seq data with DESeq2. *Genome Biol* 2014; **15**: 550.

29. Wickham H. ggplot2: Elegant graphics for data analysis. 2016. Springer-Verlag, New York.

30. Slowikowski K. Automatically position non-overlapping text labels with ‘ggplot2’. 2019. , R package version 0.8.1

31. Kassambara, Alboukadel, Mundt F. Multivariate data analyses. 2017. , R package version 1.0.5

32. Makarova K, Wolf Y, Koonin E. Archaeal clusters of orthologous genes (arCOGs): an update and application for analysis of shared features between Thermococcales, Methanococcales, and Methanobacteriales. *Life* 2015; **5**: 818–840.

33. Dulmage KA, Darnell CL, Vreugdenhil A, Schmid AK. Copy number variation is associated with gene expression change in archaea. *Microb genomics* 2018; **4**: e000210.

34. Eddy SR. Accelerated profile HMM searches. *PLoS Comput Biol* 2011; **7**: e1002195.

35. Finn RD, Bateman A, Clements J, Coggill P, Eberhardt RY, Eddy SR, et al. Pfam: The protein families database. *Nucleic Acids Res* . 2014. Oxford University Press. , **42**: D222

36. Katoh K. MAFFT: a novel method for rapid multiple sequence alignment based on fast Fourier transform. *Nucleic Acids Res* 2002; **30**: 3059–3066.

37. Nguyen LT, Schmidt HA, Von Haeseler A, Minh BQ. IQ-TREE: A fast and effective stochastic algorithm for estimating maximum-likelihood phylogenies. *Mol Biol Evol* 2015; **32**: 268–274.

38. Letunic I, Bork P. Interactive Tree Of Life (iTOL): An online tool for phylogenetic tree display and annotation. *Bioinformatics* 2007; **23**: 127–128.

39. Rutherford K, Parkhill J, Crook J, Horsnell T, Rice T, Rajandream M, et al. Artemis sequencing visualization and annotation. *Bioinformatics* 2000; **16**: 944–945.

40. Grant CE, Bailey TL, Noble WS. FIMO: Scanning for occurrences of a given motif. *Bioinformatics* 2011; **27**: 1017–1018.

41. Bagchi DN, Iyer VR. The determinants of directionality in transcriptional initiation. *Trends Genet* 2016; **32**: 322–333.

42. Anantharaman V, Koonin E V, Aravind L. Regulatory potential, phyletic distribution and evolution of ancient, intracellular small-molecule-binding domains. *J Mol Biol* 2001; **307**: 1271–1292.

43. Osman D, Cavet JS. Bacterial metal-sensing proteins exemplified by ArsR–SmtB family repressors. *Nat Prod Rep* 2010; **27**: 668.

44. Rademacher C, Masepohl B. Copper-responsive gene regulation in bacteria. *Microbiol (United Kingdom)* 2012; **158**: 2451–2464.

45. Zhang Z, Schwartz S, Wagner L, Miller W. A greedy algorithm for aligning DNA sequences. *J Comput Biol* . 2000. , **7**: 203–214

46. Morgulis A, Coulouris G, Raytselis Y, Madden TL, Agarwala R, Schäffer AA. Database indexing for production MegaBLAST searches. *Bioinformatics* 2008; **24**: 1757–1764.

47. Vallenet D, Calteau A, Dubois M, Amours P, Bazin A, Beuvin M, et al. MicroScope: an integrated platform for the annotation and exploration of microbial gene functions through genomic, pangenomic and metabolic comparative analysis. *Nucleic Acids Res* 2020; **48**: D579–D589.

48. Ott E, Kawaguchi Y, Özgen N, Yamagishi A, Rabbow E, Rettberg P, et al. Proteomic and metabolomic profiling of *Deinococcus radiodurans* after exposure simulated low Earth orbit vacuum. *Front Microbiol* 2019; **10**: 909.

49. Cox J, Mann M. MaxQuant enables high peptide identification rates, individualized p.p.b.-range mass accuracies and proteome-wide protein quantification. *Nat Biotechnol* 2008; **26**: 1367–1372.

1. CR and LH are co-first authors [↑](#footnote-ref-1)
