# Supplementary figures and images for: Genome wide transcriptomic analysis of the soil ammonia oxidizing archaeon Nitrososphaera viennensis upon exposure to copper limitation
Source: ISME J. 2020 Jul 14;14(11):2659–74. doi: 10.1038/s41396-020-0715-2 (PMC7785015; doi:10.1038/s41396-020-0715-2)

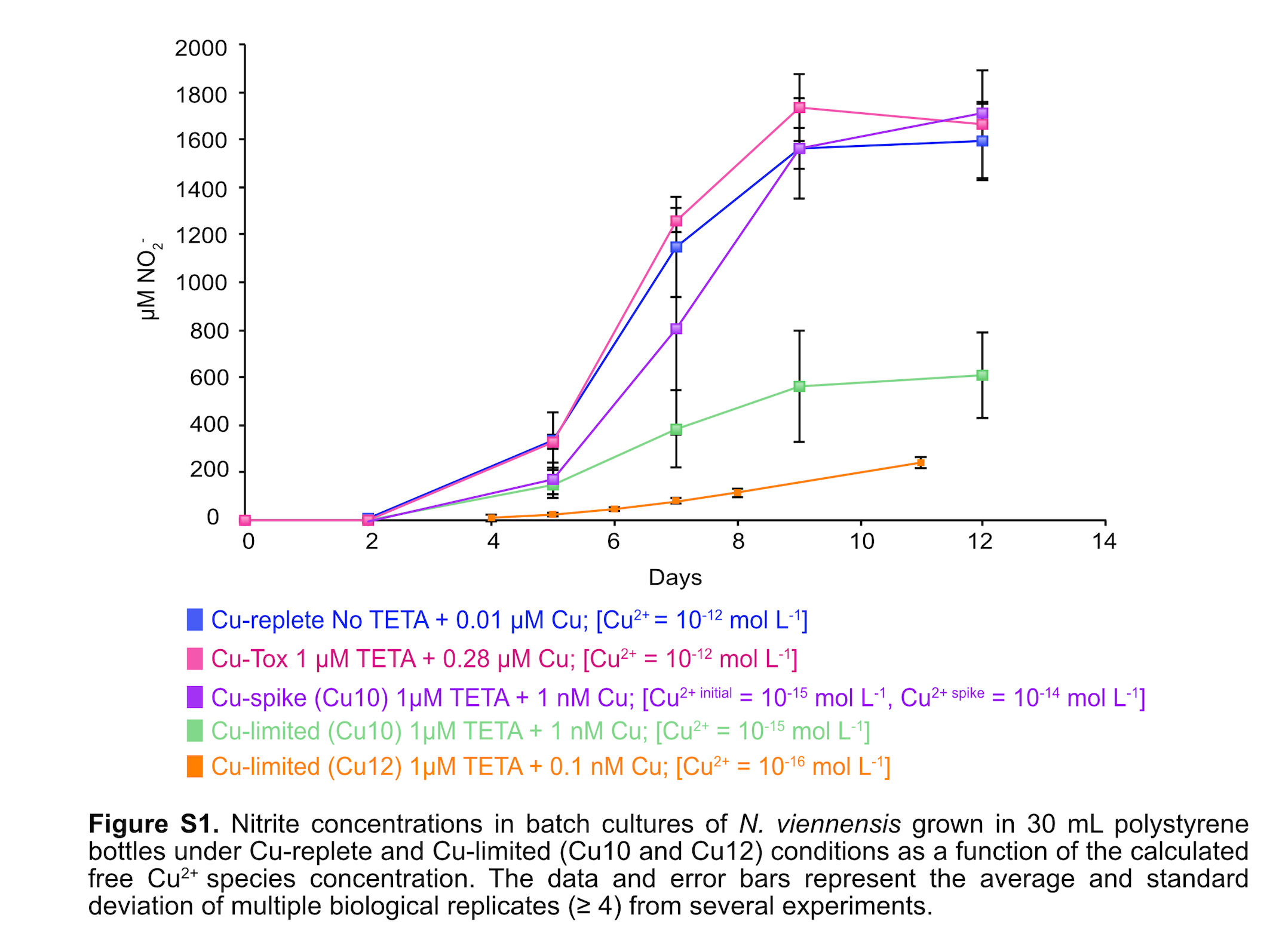

Supplement: Supplementary file 2 — Figure S1 [file 41396_2020_715_MOESM2_ESM.tif]

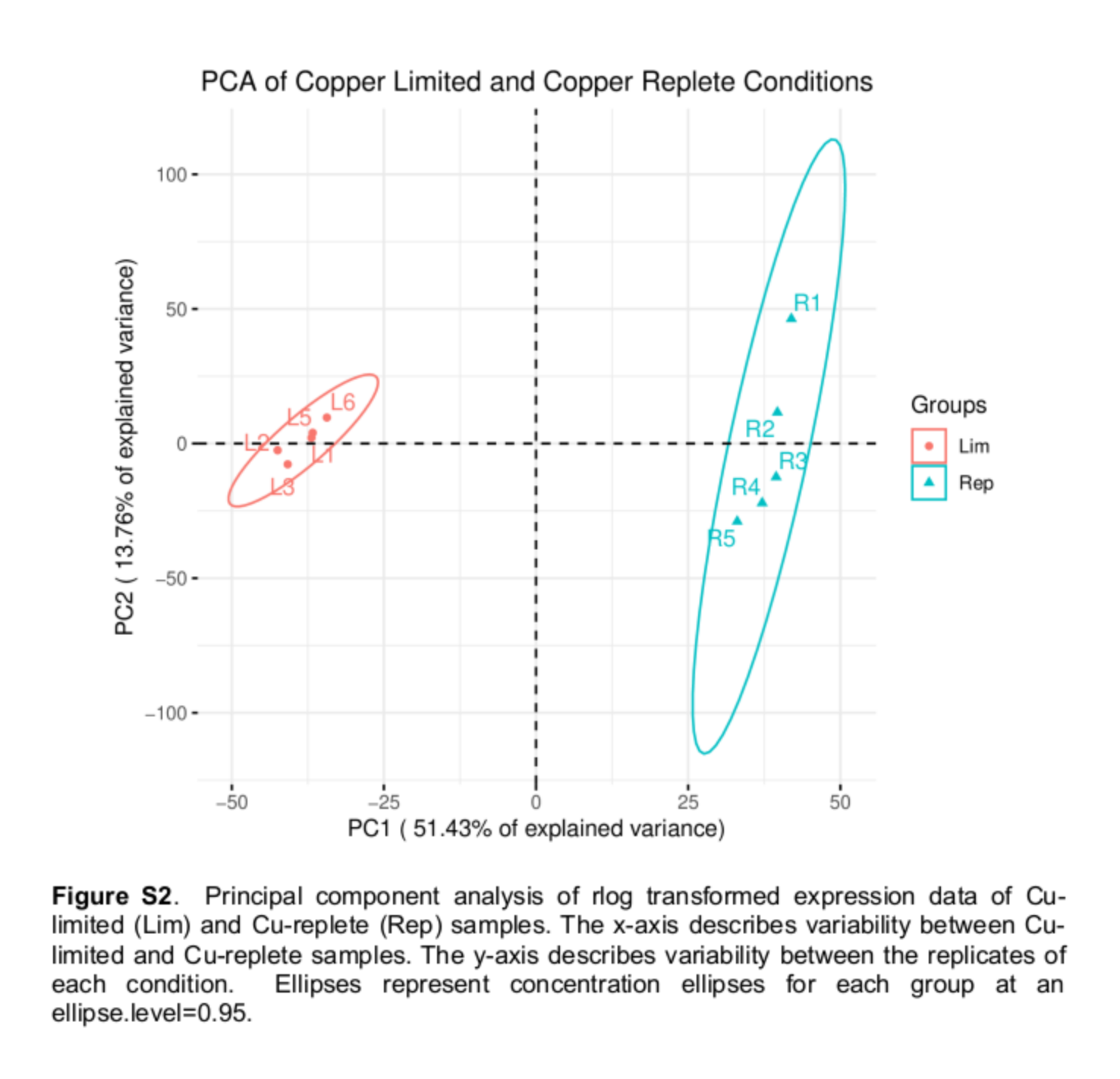

Supplement: Supplementary file 3 — Figure S2 [file 41396_2020_715_MOESM3_ESM.tiff]

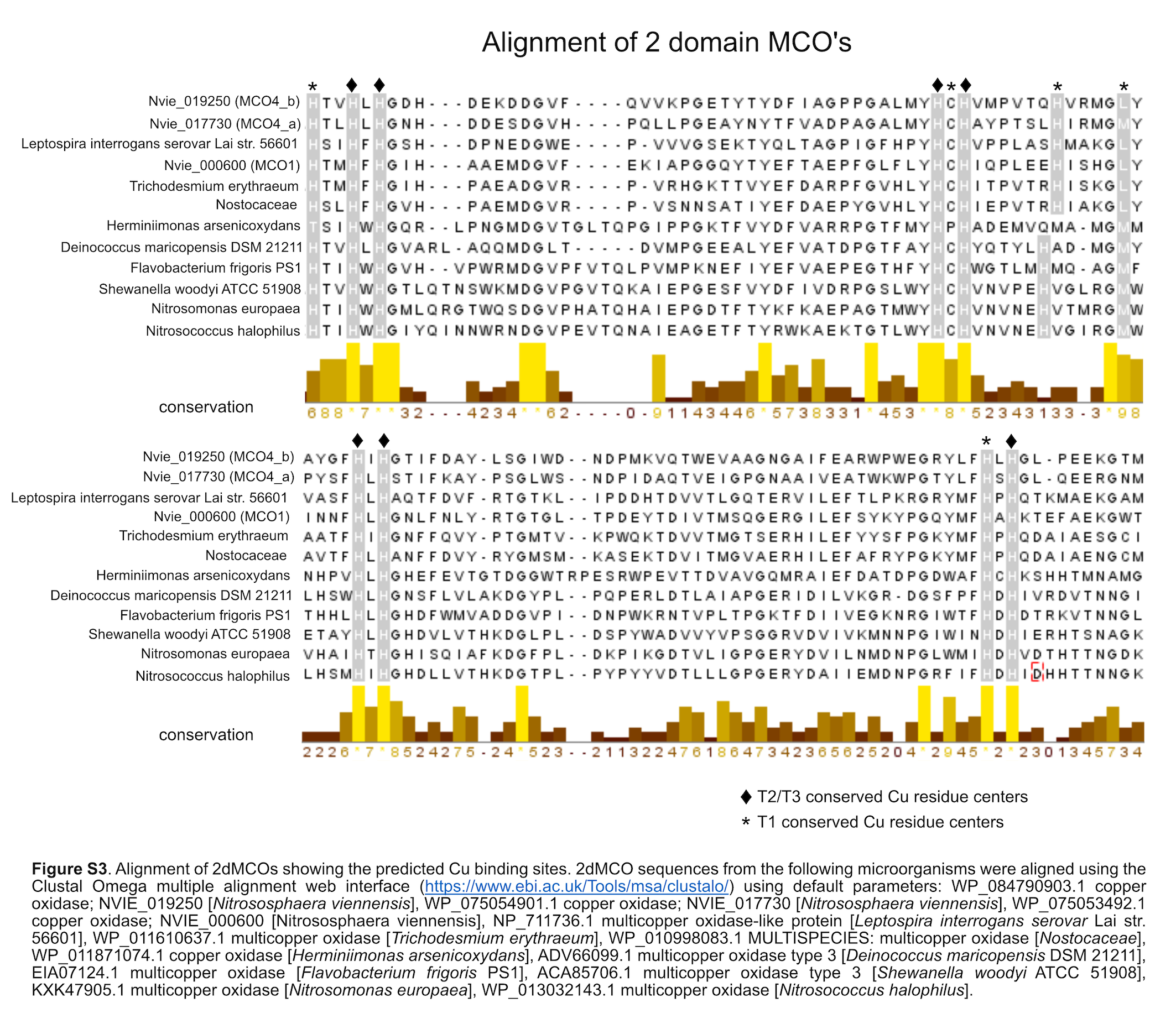

Supplement: Supplementary file 4 — Figure S3 [file 41396_2020_715_MOESM4_ESM.tif]

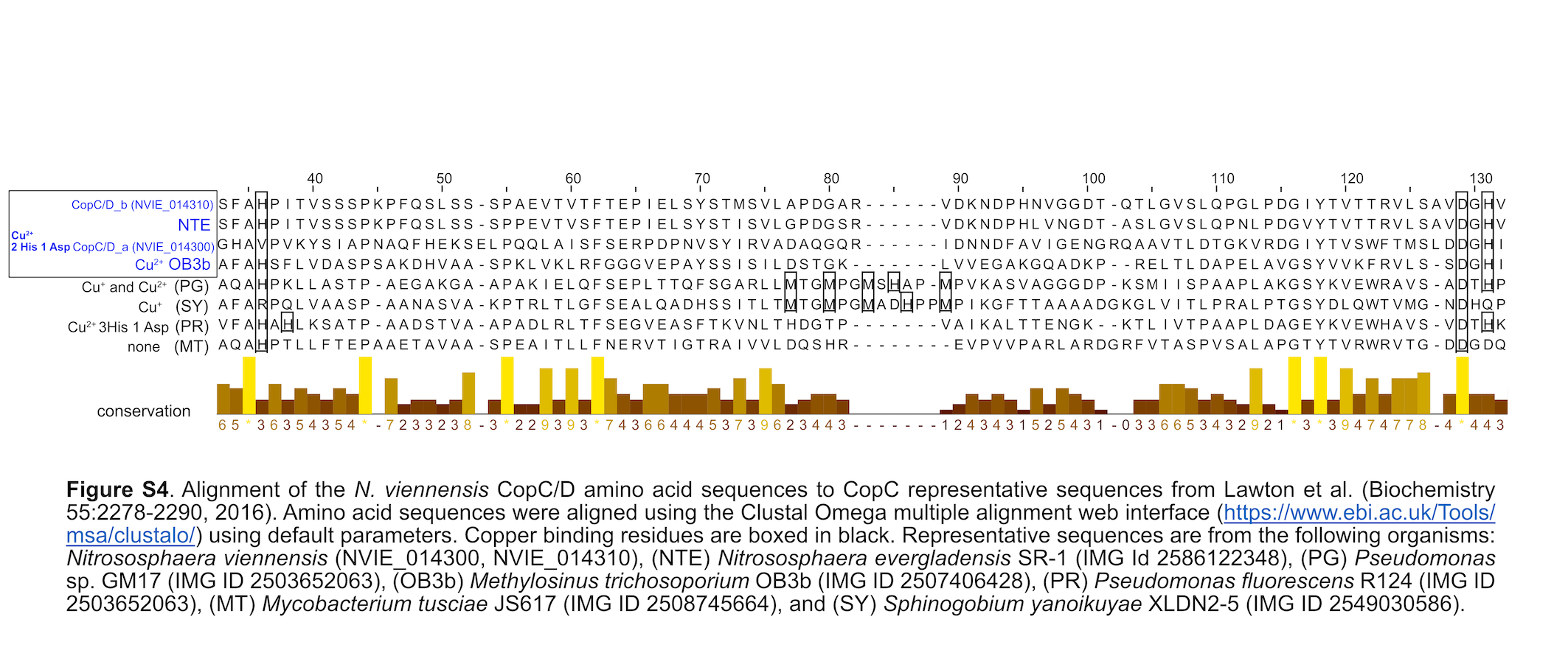

Supplement: Supplementary file 5 — Figure S4 [file 41396_2020_715_MOESM5_ESM.tif]

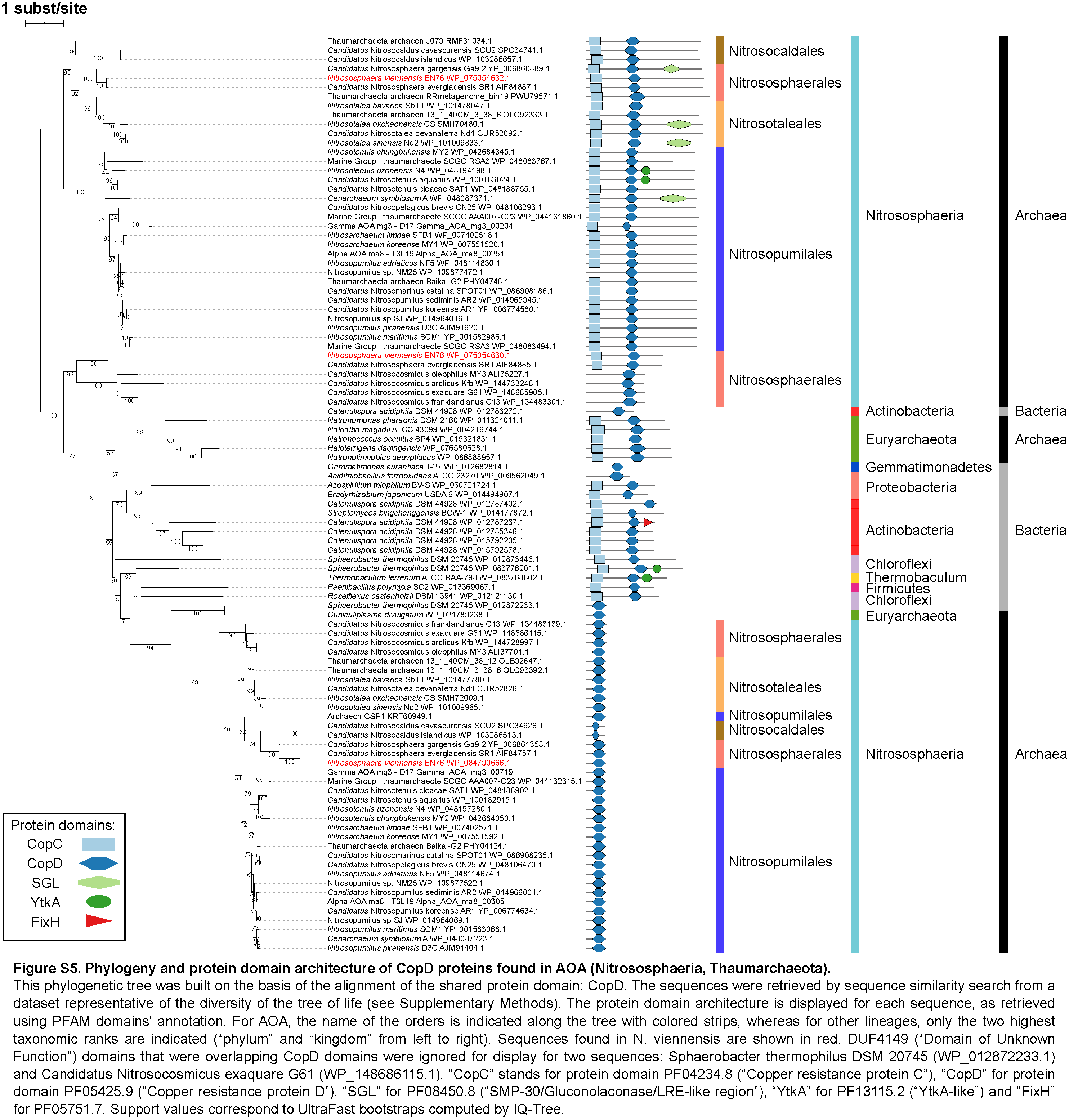

Supplement: Supplementary file 6 — Figure S5 [file 41396_2020_715_MOESM6_ESM.tif]

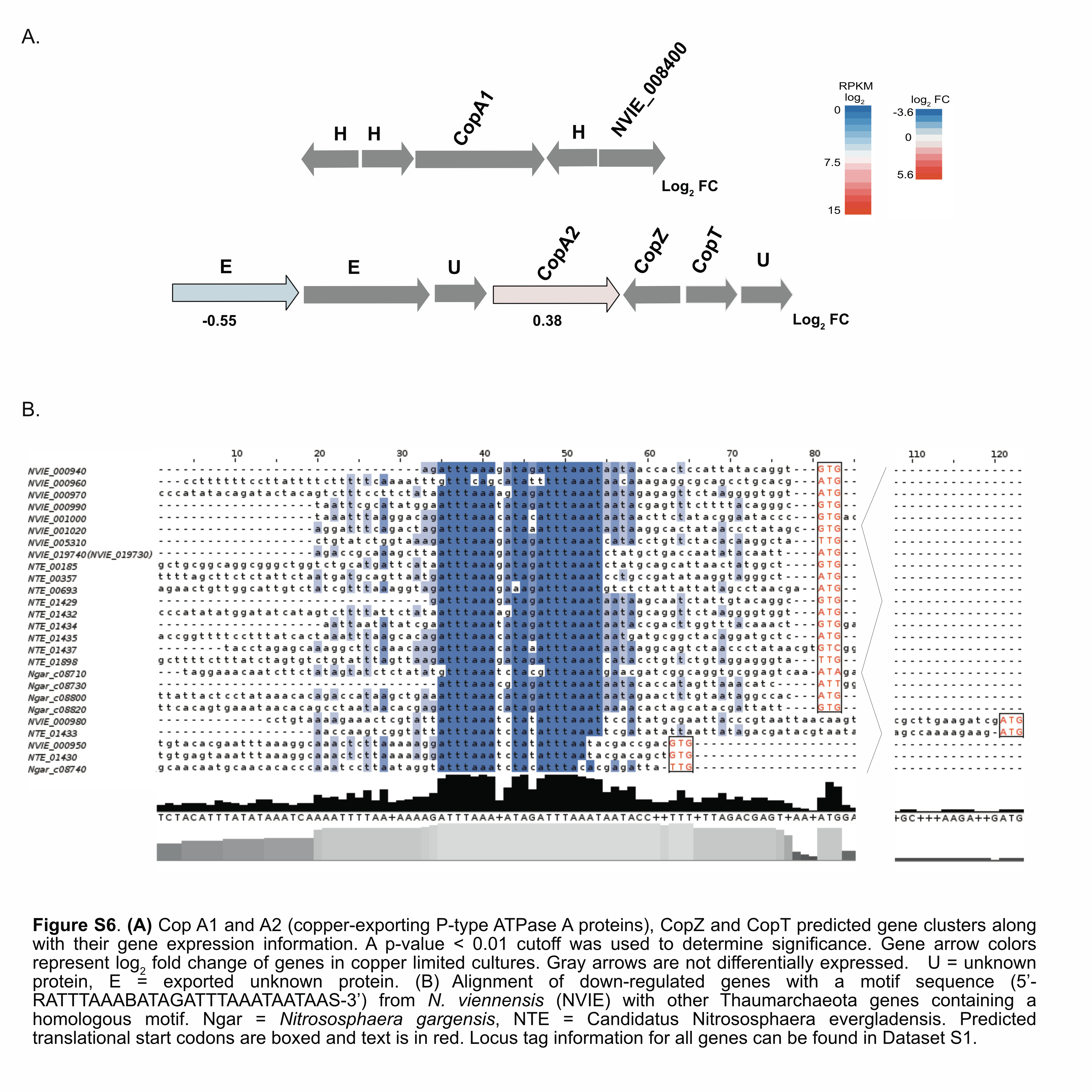

Supplement: Supplementary file 7 — Figure S6 [file 41396_2020_715_MOESM7_ESM.tif]

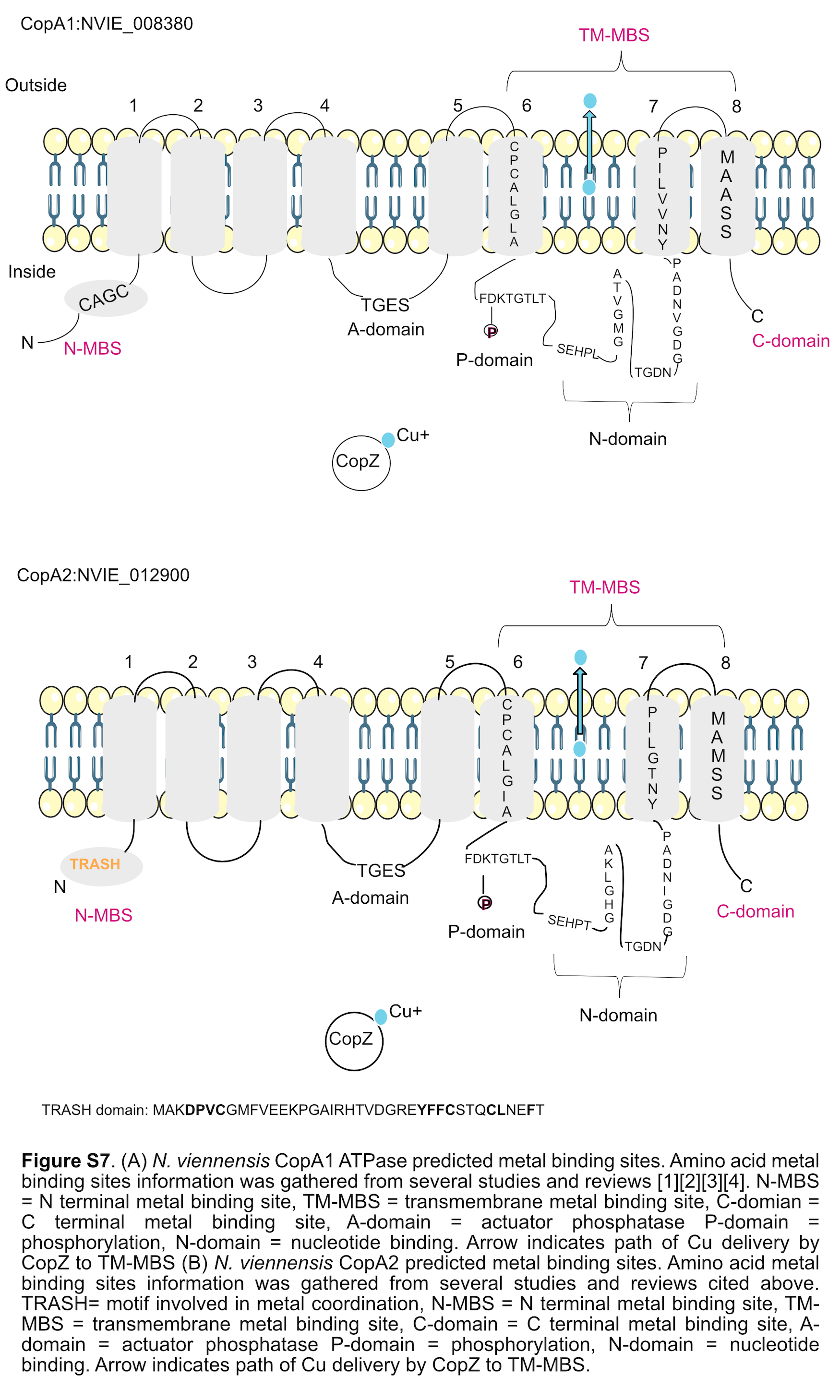

Supplement: Supplementary file 8 — Figure S7 [file 41396_2020_715_MOESM8_ESM.tif]

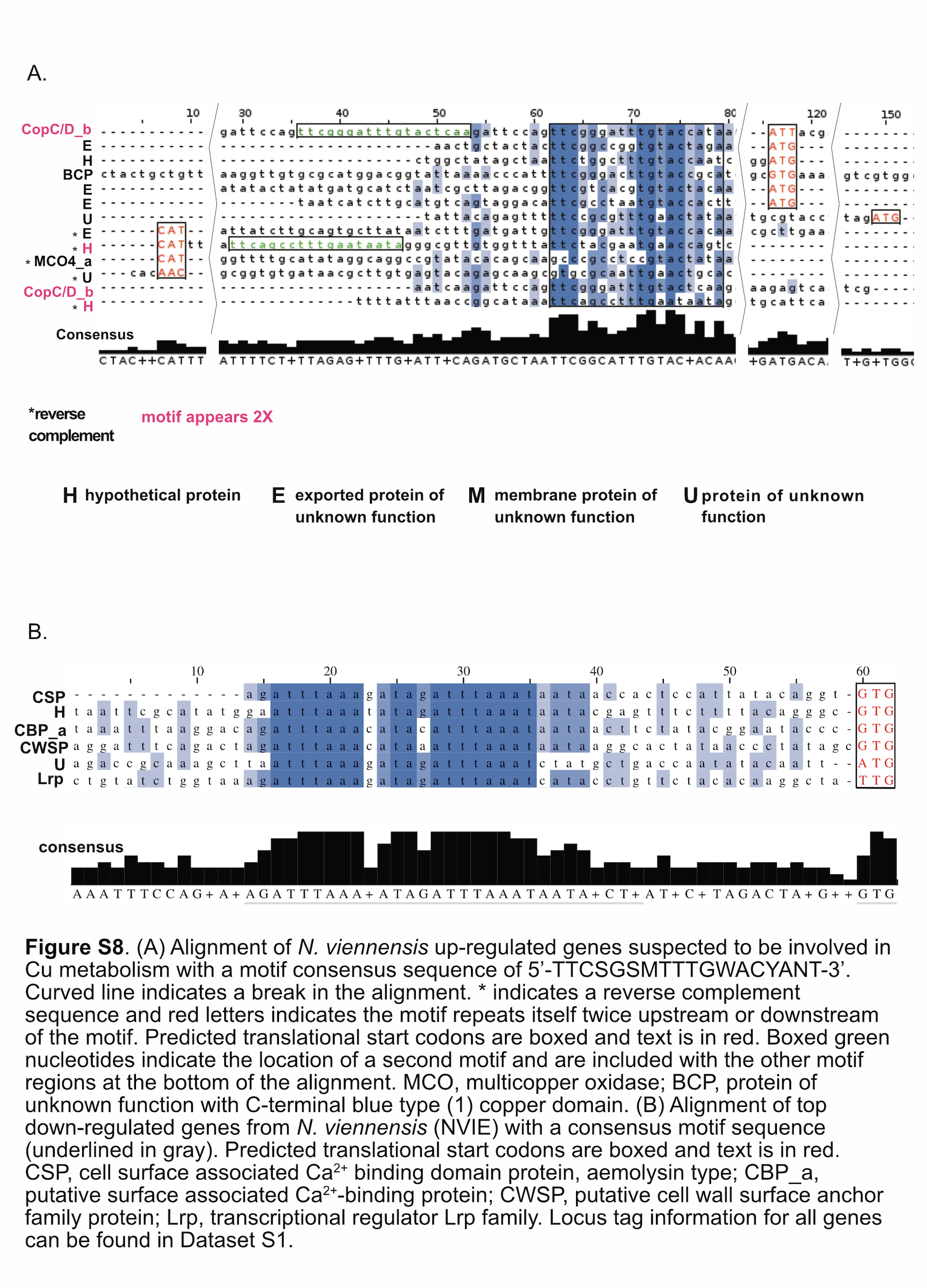

Supplement: Supplementary file 9 — Figure S8 [file 41396_2020_715_MOESM9_ESM.tif]

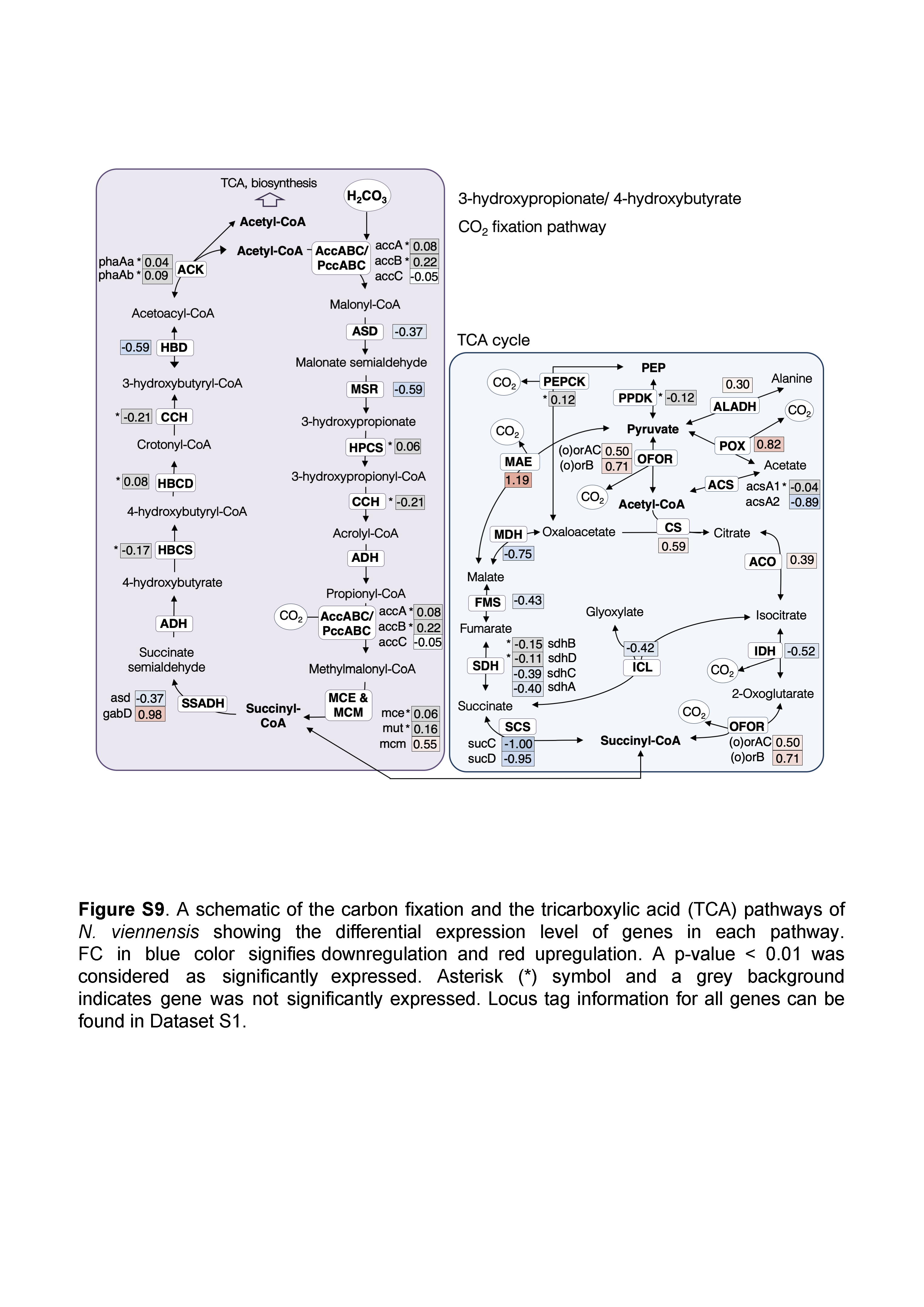

Supplement: Supplementary file 10 — Figure S9 [file 41396_2020_715_MOESM10_ESM.tif]
