# Supplementary material for: Genome wide transcriptomic analysis of the soil ammonia oxidizing archaeon Nitrososphaera viennensis upon exposure to copper limitation
Source: ISME J. 2020 Jul 14;14(11):2659–74. doi: 10.1038/s41396-020-0715-2 (PMC7785015; doi:10.1038/s41396-020-0715-2)
Supplement: Supplementary file 11 — Table S1 [file 41396_2020_715_MOESM11_ESM.docx]

**Table S1. RNA-Seq results summary of samples that were sequenced.**

| **Sample name** | *L1Day7 | *L2Day7 | *L3Day7 | *L5Day7 | *L6Day7 | †R1Day5 | †R2Day5 | †R3Day5 | †R4Day5 | †R5Day5 |
| --- | --- | --- | --- | --- | --- | --- | --- | --- | --- | --- |
| ****Trimmed** | 36,290,278 | 47,566,588 | 38,440,347 | 62,002,562 | 58,389,026 | 37,012,985 | 34,328,560 | 42,107,829 | 36,910,739 | 36,423,799 |
| **‡ rRNA sequences** | 34,671,567 | 45,422,238 | 36,818,224 | 58,629,182 | 55,664,484 | 37,753,008 | 32,4191,20 | 39,749,355 | 34,829,426 | 36,423,799 |
| **#Non-rRNA sequences** | 1,618,711 | 2,144,350 | 1,622,123 | 3,373,380 | 2,724,542 | 2,259,977 | 1,909,440 | 2,358,474 | 2,081,313 | 1,672,974 |
| **‡‡Sequences assigned to a genomic feature** | 1,515,278 | 1,988,513 | 1,497,003 | 3,189,422 | 2,523,110 | 2,138,075 | 1,792,743 | 2,210,469 | 1,949,417 | 1,568,005 |
| **+Unassigned Sequences** | 103,433 | 155,837 | 125,120 | 183,958 | 201,432 | 121,902 | 116,697 | 148,005 | 131,896 | 104,969 |

*Cu-limited cultures

† Cu-replete cultures

** remaining sequences after adaptor and low quality sequences were trimmed away from raw sequences

‡ number of sequences matching rRNA genes

# number of sequences not matching rRNA reads used for genomics feature assignment

‡‡ number of sequences that could be assigned to genes in the reference genome

+ number of sequences that could not be assigned to genes in the reference genome
