# Supplementary material for: Genome wide transcriptomic analysis of the soil ammonia oxidizing archaeon Nitrososphaera viennensis upon exposure to copper limitation
Source: ISME J. 2020 Jul 14;14(11):2659–74. doi: 10.1038/s41396-020-0715-2 (PMC7785015; doi:10.1038/s41396-020-0715-2)
Supplement: Supplementary file 12 — Table S2 [file 41396_2020_715_MOESM12_ESM.docx]

Table S2. Primers used in qPCR.

| Gene | Primer name | Forward primer 5’ to 3’ | Reverse primer 5’ to 3’ | Source or reference |
| --- | --- | --- | --- | --- |
| Zn-Fe permease  PERM_b  NVIE_000590 | PERM1 | GGCAGCAGGACTACGACGACA | GCCCTCGGTGGTGTTGTGTATC | This study |
| Multicopper oxidase  MCO1  NVIE_000600 | MCO1 | CGCTGAAAAACGCCGTGGTG | CACGAGGTCGCCTTCCGTTG | This study |
| Putative Ca^2+^ binding protein  CBP_a  NVIE_001000 | qNV001000 | TGTTCCCCACTCGTCTACCACC | ATTGGATCCGACGGCAACAC | This study |
| Multicopper oxidase  MCO4_b  NVIE_019250 | qNV-1019250 | GCGCGATATTTGAGGCCAGA | TTGTCGCTCCGGAAGGATCT | This study |
| *16S rRNA* | qNV16s | GAGTGGGCACTGAGACAAGGG | CGGCTACGGATGCTTTAGGC | This study |
| *rpoB* | qrpoB | CATAATCCCGTACCCCGAGC | CGCCTTCGTGGAAACAACAG | This study |
